# Treating Semiempirical Hamiltonians as Flexible Machine Learning Models Yields Accurate and Interpretable Results

**DOI:** 10.1021/acs.jctc.3c00491

**Published:** 2023-09-14

**Authors:** Frank Hu, Francis He, David J. Yaron

**Affiliations:** Department of Chemistry, Carnegie Mellon University, Pittsburgh, Pennsylvania 15213, United States

## Abstract

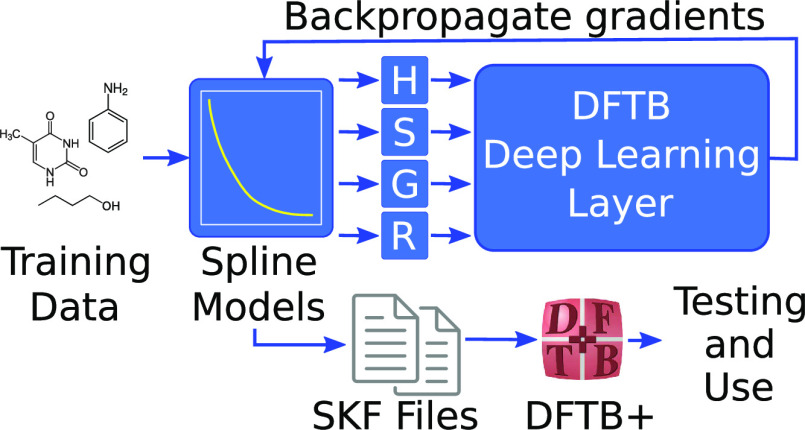

Quantum chemistry provides chemists with invaluable information, but the high computational cost limits the size and type of systems that can be studied. Machine learning (ML) has emerged as a means to dramatically lower the cost while maintaining high accuracy. However, ML models often sacrifice interpretability by using components such as the artificial neural networks of deep learning that function as black boxes. These components impart the flexibility needed to learn from large volumes of data but make it difficult to gain insight into the physical or chemical basis for the predictions. Here, we demonstrate that semiempirical quantum chemical (SEQC) models can learn from large volumes of data without sacrificing interpretability. The SEQC model is that of density-functional-based tight binding (DFTB) with fixed atomic orbital energies and interactions that are one-dimensional functions of the interatomic distance. This model is trained to *ab initio* data in a manner that is analogous to that used to train deep learning models. Using benchmarks that reflect the accuracy of the training data, we show that the resulting model maintains a physically reasonable functional form while achieving an accuracy, relative to coupled cluster energies with a complete basis set extrapolation (CCSD(T)*/CBS), that is comparable to that of density functional theory (DFT). This suggests that trained SEQC models can achieve a low computational cost and high accuracy without sacrificing interpretability. Use of a physically motivated model form also substantially reduces the amount of *ab initio* data needed to train the model compared to that required for deep learning models.

## 1 Introduction

A substantial challenge for quantum chemistry is lowering the computational cost^1−6^ to enable accurate predictions on large systems such as those of interest in biological and material applications. Molecular systems have two properties that provide the basis for approximations that lower computational costs: nearsightedness and molecular similarity. Nearsightedness provides the chemical basis for methods that have, over the past few decades, substantially reduced computational costs without large sacrifices in accuracy. In particular, large reductions in cost can be achieved by replacing detailed Coulombic interactions, required at short range, with increasingly coarse-grained multipolar interactions at long range.^7−11^ Methods have also been developed that use molecular similarity to achieve dramatic reductions in computational cost, including molecular mechanics^12,13^ and semiempirical quantum chemistry (SEQC).^14,15^ Unfortunately, these cost reductions have typically come with a substantial decrease in accuracy. More recently, machine learning (ML) has emerged as a means to leverage molecular similarity to develop models that are both low-cost and accurate.^16−20^ However, current applications of ML in chemistry often incorporate little physics and function as black boxes that are difficult to interpret. Here, we combine ML with SEQC to create physics-based models that achieve high accuracy and computational efficiency without sacrificing interpretability.

The ability of ML to leverage molecular similarity stems from the use of highly flexible model forms such as the artificial neural networks (NNs)^21−28^ of deep learning. This flexibility enables ML models to learn from large volumes of training data. For example, the accuracy of the ANI-1 neural network potential^22^ improves as it shows more training data, approaching chemical accuracy^29−33^ of 1 kcal/mol when trained to *ab initio* results on millions of molecular configurations. However, the flexibility of ML models is a double-edged sword. It leads to high accuracy, but it also makes it difficult to gain insight into the physical or chemical basis for the predictions.

SEQC provides alternative model forms that are capable of learning from data. Traditional SEQC model forms such as PM3^34^ only have a handful of parameters and this limits their ability to take advantage of large volumes of data.^35^ Replacing these single parameters with NNs imparts the flexibility to learn from large volumes of data;^36^ however, the NNs function as black boxes and so decrease interpretability. Here, we increase the flexibility of SEQC models so that they can take advantage of larger volumes of data while retaining a purely physics-based form. This is operationalized using the density-functional-based tight binding (DFTB)^37,38^ Hamiltonian with model parameters that can be expressed in the Slater–Koster File (SKF) format.^39^ DFTB includes only valence electrons and uses a minimal atomic orbital basis. The atomic orbitals are assumed to be fixed and, so, have no dependence on the molecular geometry. As a result, the atomic orbital energies are constants, and the interactions and overlaps between atomic orbitals are one-dimensional functions of the interatomic distance. These constants and one-dimensional functions are adjusted during training. We will refer to this as the SKF-DFTB model form and to our resulting trained models as DFTBML.

The flexibility of DFTBML lies primarily in the one-dimensional functions. Over the distances present in typical molecules, the interactions described by these functions vary by hundreds of kcal/mol. Because the molecular energy arises from many such interactions, changes of a few tenths of kilocalories per mole can have significant effects on the total energy. For the model to learn effectively from data, we need a functional form with the sensitivity to fine-tune these interactions while preventing oscillations and other nonphysical behaviors. Here, flexibility and sensitivity are provided through splines, i.e., piecewise polynomials, with a high polynomial order of five and a large number of 100 knots. To prevent oscillations and other nonphysical behaviors, a strong regularization scheme is developed and implemented in our training of DFTBML.

In this study, we train and evaluate the DFTBML model as one would for any other machine learning model. The approach thereby shares the limitations that result from the use of a specific set of training data. The training and testing data used here are for organic molecules with closed shell electronic configurations and whose structures are distorted from their minimum energy geometry. This allows us to evaluate the performance of a physics-based, interpretable model on tasks that are well-studied for models that incorporate black-box components.

**Figure 1:**
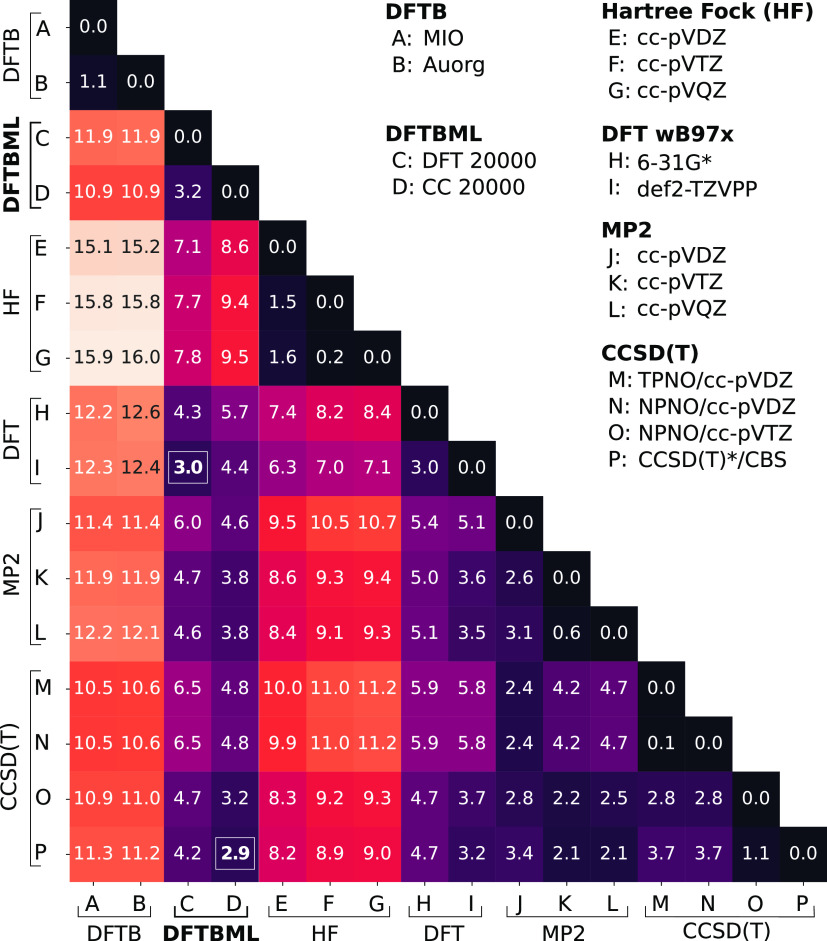
Comparison of different quantum chemistry methods on atomization energies (eq 2 and Section 4). The heatmap is generated from the ∼230 k molecular configurations in the ANI-1CCX data set with up to eight heavy atoms, after removing configurations with incomplete entries. The DFTBML DFT/CC parametrizations were trained to wB97x/def2-TZVPP or CCSD(T)*/CBS energies, respectively, on 20 000 molecules with up to eight heavy atoms. Agreement of DFTBML with the method to which it was trained is highlighted in white boxes. DFTBML improves substantially on currently published DFTB parameters (MIO^37^ and Auorg^40^), with the agreement between DFTBML CC and CCSD(T)*/CBS being somewhat better than that between DFT (wB97x/def2-TZVPP) and CCSD(T)*/CBS.

The DFTBML models explored here are trained to the ANI-1CCX data set,^25^ which includes results from a number of different *ab initio* methods on organic molecules comprised of C, N, O, and H. The DFTBML models can reproduce the predictions of CCSD(T)*/CBS to about 3 kcal/mol, which is comparable to the accuracy of DFT (see Figure 1). We also show that 20 000 molecular configurations are sufficient to train the model. This saturation of performance with increasing data suggests that the accuracy is limited by the SKF-DFTB model form itself and not by the amount of training data. The data requirements of DFTBML are considerably below the ∼1 M data points typically used to train deep learning models, which is significant given that the generation of *ab initio* training data is a primary computational bottleneck in model development. This opens the possibility of using trained SEQC models as replacements for DFT, substantially reducing computational cost without, as in traditional SEQC models, sacrificing accuracy or, as in many ML models, sacrificing interpretability.

## 2 Results and Discussion

### 2.1 Model Form

**Figure 2:**
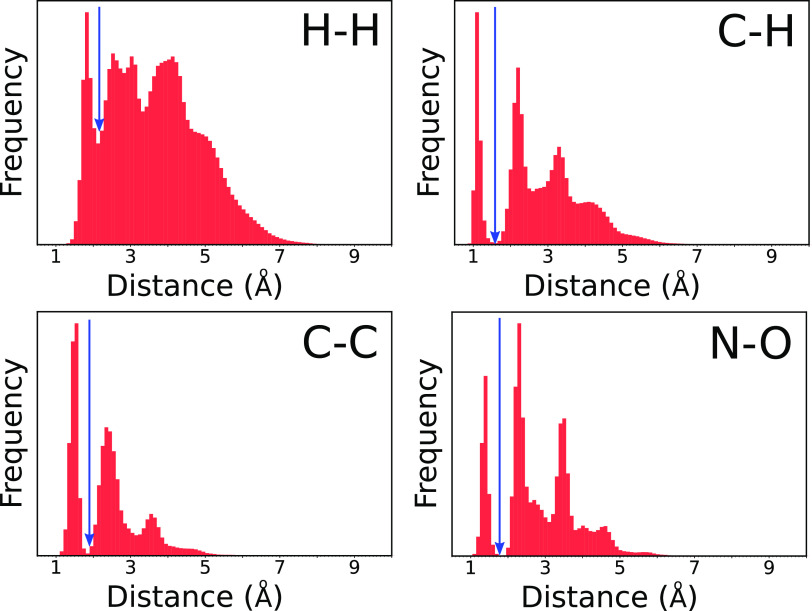
Distributions of internuclear distances between H–H, C–C, C–H, and N–O in the cleaned ANI-1CCX data set for molecules with up to eight heavy atoms. Repulsive interactions are truncated beyond nearest-neighbor interactions (blue arrows) with a lower bound of 0 Å. Electronic interactions go to a longer range (4.5 Å) with a lower bound slightly lower than the shortest distance in a given distribution. Precise cutoffs for electronic and repulsive splines can be found in Tables S2 and S3, respectively.

The DFTBML model developed here uses a Hamiltonian that is similar in form to that of DFTB, with the distinction being in the approach used to obtain the model parameters. DFTB uses a physics-based procedure to derive Hamiltonian matrix elements for a valence-only minimal atomic basis set. *Ab initio* data on molecules are used only to determine an empirical pairwise-additive repulsive potential that accounts for interactions between core electrons not included in the electronic Hamiltonian. Here, we instead fit all aspects of the model to *ab initio* data while retaining the following restrictions imposed by the SKF file format: the atomic orbital energies are trained constants; the one-electron Hamiltonian matrix elements (**H**_**1**_), overlap integrals (**S**), and repulsive potentials (**R**) are functions of only interatomic distance; Coulombic interactions (**G**) use a model form that depends only on Hubbard parameters associated with the atomic shells.^37^ We use fifth-order splines for the electronic (Hamiltonian and overlap) functions and third-order splines for the repulsive potentials, with distance ranges specified by analyzing distributions of pairwise distances (see Figure 2). Boundary conditions are applied only at the upper limit, where we force both the function and its derivative to go to zero at large interatomic separations. No boundary conditions are imposed at the lower limit. The model is implemented in PyTorch^41^ in a manner that is optimized specifically for training of model parameters. In a precompute phase, information that is independent of the model parameters is computed and stored for all molecules used in the training process. During training, these data are loaded and used to compute model predictions and associated gradients. Additional details are provided in Section 4 and in the Supporting Information.

In addition to retaining a physics-based and interpretable model form, SKF-DFTB has the advantage that trained models can be easily distributed through SKF files that are supported by many computational chemistry packages.^13,39,42^ All test results quoted here were obtained from DFTB+ using SKF files produced by our training code. This approach gives a stronger guarantee of the validity of the quoted model performances.

### 2.2 Experimental Design

**Figure 3:**
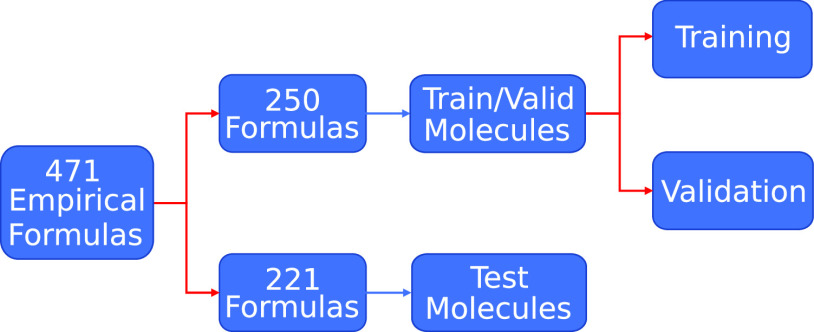
Overview of the method used to generate data sets. Red arrows indicate random sampling. Molecules are divided based on their empirical formulas, ensuring no mixing between training and testing data.

For developing DFTBML, we use the ANI-1CCX data set,^25^ which contains organic molecules with elements C, N, O, and H. We focus here only on molecules with up to eight heavy (nonhydrogen) atoms and we retain only configurations that have complete entries for all fields, resulting in 471 unique empirical formulas with a total of 232 310 molecular configurations. The division of the data into training, validation, and testing data is schematically shown in Figure 3. We divide molecules by empirical formulas to ensure that there is no overlap between training and testing data. A more detailed explanation can be found in Section S10.

To explore the performance of DFTBML, we trained the model under various conditions. To aid comparisons, it is useful to introduce a standard notation for the resulting parameter sets. To evaluate the generalization of the DFTBML models, we consider both near- and far-transfer, with the difference being the degree to which the model is being transferred to larger systems. For near-transfer, where the training and testing data contain systems with 1–8 heavy atoms, we use “DFTBML” followed by the energy target (DFT for wB97x/def2-TZVPP; CC for CCSD(T)*/CBS) and the number of configurations in the training set, e.g., “DFTBML CC 20000”. For far-transfer, where the training data have molecules with 1–5 heavy atoms, while the test data have molecules with 6–8 heavy atoms, we use “Transfer” as the prefix, e.g., “Transfer CC 20000”. We also consider results obtained when only a short-range repulsive potential is trained to the data, with the electronic parameters being those of Auorg.^40^ For these models, we use “Repulsive” as a prefix, e.g., “Repulsive CC 20000”.

The optimization is done on an L2 loss
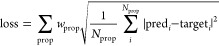
1where *N*_prop_ is the number of instances in the training data set and *w*_prop_ is the weight (defaulting to 6270 Ha^–1^ for total molecular energy per heavy atom, 100 (eÅ)^−1^ for Cartesian dipole components, and 1 e^–1^ for atomic charges). The total energies are for the DFT and CC targets, as described above. Because the DFTBML model includes a reference energy term that allows the energy of isolated atoms to be optimized, the energy component of the loss function is based on the difference between the predicted and target atomization energies (see Section 4). The effects of changing the relative weighting of energy and dipole are explored below along with the rationale used to choose the default weights. The weight for charges was chosen to be sufficiently small that it does not impact the performance on the other properties. Our emphasis on dipoles versus charges is partly motivated by the dipole being an experimental observable, whereas atomic charges can vary with the approach used to assign charges. Here, the targets are CM5^43^ charges from wB97*x*/6-31G* because these are available in the ANI-1CCX data set. Note that wB97*x*/6-31G* dipoles and charges are used for the DFT and CC energy targets. The atomic charges and dipoles of the DFTBML model are obtained from the charge fluctuations in the DFTB Hamiltonian.^37^ The loss also includes the regularization penalties discussed in the next section.

### 2.3 Effects of Regularization on the Model Performance

**Figure 4:**
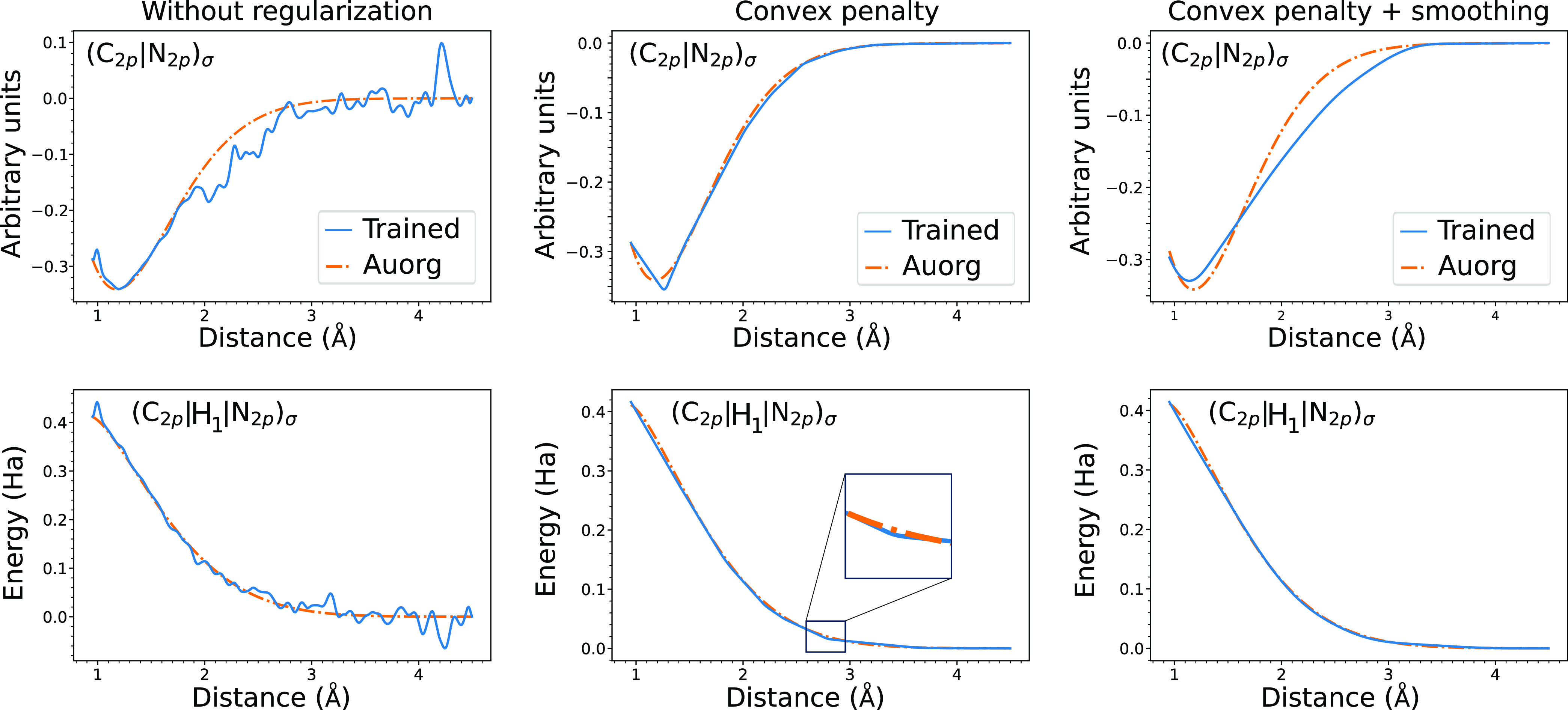
Effects of regularization on (C_2*p*_|*N*_2*p*_)_σ_ overlaps (**S**, top row) and Hamiltonian elements (**H**_**1**_, bottom row): no regularization (left column), convex penalty that constrains the sign of the second derivative (middle column), and convex plus smoothing that penalizes the magnitude of the third derivative (right column). The Auorg reference functions (orange, dashed lines) are included for comparison to the functions trained on the Transfer CC 2500 data set (blue).

A challenge with developing the DFTBML model was creating an effective regularization scheme that would prevent overfitting without degrading the model performance by being too restrictive. Without regularization, the resulting functions show highly oscillatory behavior (left column of Figure 4). Previous works^36,44^ penalized deviations from a set of physically derived reference parameters, e.g., deviation from the Auorg parameter set of DFTB. This approach to regularization is problematic because it may overly bias the training toward the reference parameters and does not prevent nonphysical behaviors such as oscillation of a trained function around the smooth form of the reference function.^44^ A commonly used approach for smoothing splines applies a penalty to the magnitude of the second derivative.^45,46^ However, for DFTBML, such a smoothing penalty substantially degrades performance of the models because there is no reason to expect the second derivative to have a limited magnitude.

We instead adapt an approach from Akshay et al.,^47^ which is motivated by the shape of the functions in reference parameter sets, such as those of Auorg in Figure 4. For the Hamiltonian (**H**_**1**_) matrix elements, the functions decay smoothly to zero and have an upward curvature. To enforce this behavior, we apply a “convex” penalty that enforces the second derivative of the trained potentials, evaluated on a dense grid of 500 points, to have a physically motivated sign (upward curvature for functions, as in the lower panels of Figure 4, that approach zero from above and downward curvature for functions approaching zero from below). For overlaps (**S**), the regularization is extended to allow for an inflection point. This is done because MIO and Auorg, which use a physics-based procedure to obtain the Hamiltonian and overlap functions, find a single inflection point in the overlap for certain pairs of atomic orbitals (top panels of Figure 4). We therefore extend the convex penalty for overlaps to allow a single inflection point, whose location is optimized during training. The inflection point is associated with nodes in the atomic orbitals and, for higher energy valence orbitals than the 2s and 2p orbitals of the first row elements studied here, it may be necessary to allow multiple inflection points. The results indicate that although inclusion of an inflection point improves model performance, the results are not sensitive to its precise location (see Section S12.3). The magnitude of the weighting factor for these convex penalties does not require fine-tuning beyond being large enough to prevent violations of the constraints without being so large that it leads to numerical instabilities in gradient descent optimization. The convex penalty successfully removes the oscillatory behavior (middle column of Figure 4). However, the resulting functions exhibit nonphysical, piecewise-linear behavior, which is more pronounced in the overlap integrals but also present in the Hamiltonian matrix elements (see inset in Figure 4).

To remove this piecewise-linear behavior, we apply a “smoothing” penalty to the third derivative based on the sum of squares of the third derivative evaluated on a grid of 500 points. Our use of a fifth-order spline for **H**_**1**_ and **S** is motivated by the high order needed for the spline to have a continuous third derivative. The magnitude of the penalty is adjusted to remove the piecewise-linear behavior while minimizing degradation of the model performance (see Section S12.4). The short-range repulsion (**R**) does not exhibit piecewise-linear behavior, so a smoothing penalty is not applied, and we use a third-order spline for **R**.

It is somewhat surprising, given the highly nonphysical behavior observed without regularization, that the effects of regularization on model performance are not more dramatic (Table 1). For near-transfer, the performance of the unregularized model (4.97 kcal/mol) is a factor of 2 better than that of the Auorg reference model (10.55 kcal/mol). This is despite the highly oscillatory behavior of the functions and the fact that the test and training data have molecules with disjoint empirical formulas. This suggests coupling between potentials, with oscillations in one potential canceling out the effects of oscillations in another potential. The effects of regularization are more pronounced for far-transfer, but even here the performance of the unregularized model (10.08 kcal/mol) is comparable to that of the Auorg reference model (11.81 kcal/mol).

**Table 1:** Effects of Regularization on Near-Transfer (DFTBML CC 2500), i.e., Training and Testing on Molecules with up to Eight Heavy Atoms, and Far-Transfer (Transfer CC 2500), i.e., Training on Molecules with 1–5 Heavy Atoms and Testing on Molecules with 6–8 Heavy Atoms

parameterization	MAE energy (kcal/mol)	MAE dipole (eÅ)	MAE charge (e)
near-transfer: DFTBML CC 2500			
Auorg	10.55	0.079	0.085
MIO	10.69	0.079	0.085
no regularization	4.97	0.037	0.056
convex only	3.17	0.041	0.060
convex with smoothing	2.95	0.036	0.054
far-transfer:Transfer CC 2500			
Auorg	11.81	0.089	0.088
MIO	11.86	0.089	0.088
no regularization	10.08	0.049	0.061
convex only	4.70	0.051	0.064
convex with smoothing	4.83	0.051	0.065

It is also noteworthy that although addition of the convex penalty leads to substantial improvements in model performance on test data, addition of the smoothing penalty has a much smaller effect and even slightly degrades performance for the far-transfer experiments. Hence, based on the typical approach of ML, where regularization is adjusted to optimize performance on test data, the smoothing penalty would not be viewed as necessary. However, the resulting functions of Figure 4 suggest that a smoothing penalty is needed to obtain physically reasonable functional forms. This illustrates a general finding of this work, that the level of regularization needed to achieve a physically reasonable model goes beyond that needed to achieve good transfer^48−51^ from train to test data.

### 2.4 Model Performance

**Figure 5:**
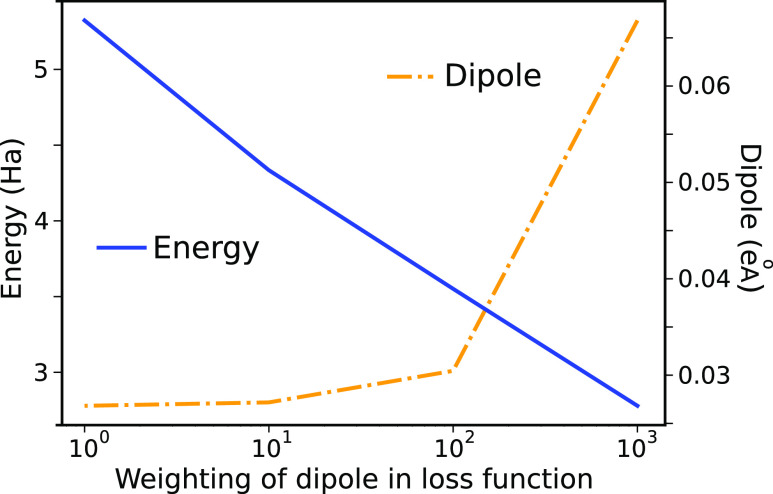
Trade-off between MAE in total energy and dipoles as a function of the dipole weighting factor for DFTBML CC 2500. A weighting factor of 100 (eÅ)^−1^ was chosen to improve the performance on dipoles while only marginally impacting the performance on total energy. More details on hyperparameter sensitivities can be found in Section S12.

By changing the weights applied to the energy and dipole components of the loss function, we can also explore the trade-off between fitting these properties. As the weight applied to the dipole component increases, we initially see large improvements in the dipole with little impact on the energy. However, beyond a weight of 10^2^, the error for energy increases rapidly (see Figure 5). For all reported results, we use a dipole weighting factor of 100 (eÅ)^−1^.

**Figure 6:**
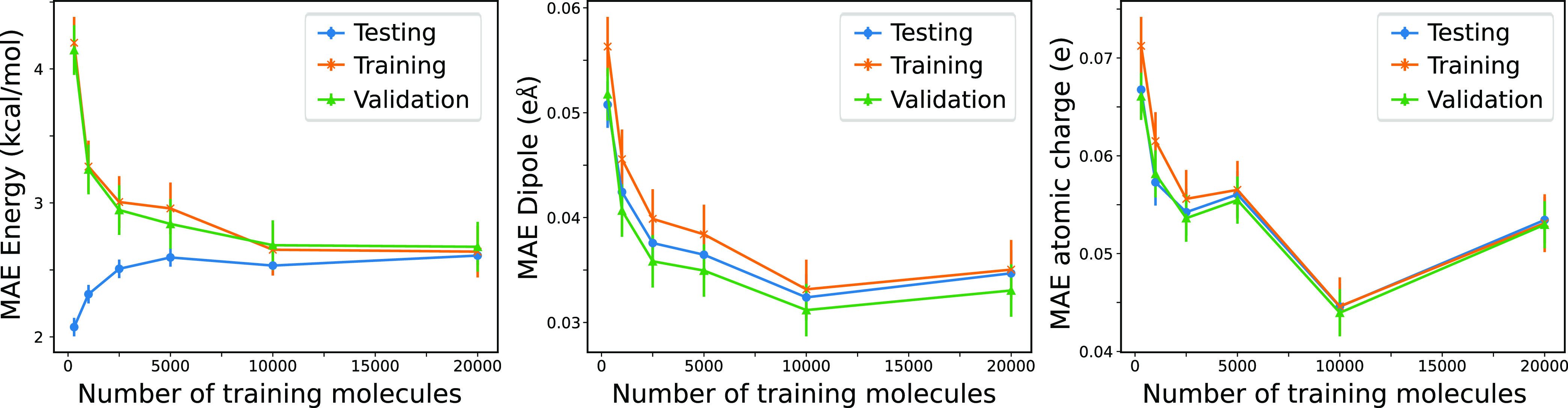
Final training, validation, and testing losses for each of the physical targets as a function of the size of the data set used for training. Results are for training to the CC energy target. Error bars are shown as  where σ is the standard deviation of the errors calculated separately for the training, validation, and testing values.

To examine how the performance of DFTBML varies with the amount of training data, models were trained on data sets with between 300 and 20 000 molecular configurations (Figure 6 and Table 2). Each model was assessed against a standard set of 10 000 test molecules. More complete results are provided in Section S13, including results from training to both CC and DFT targets and learning curves for each experiment.

**Table 2:** Performance of Various Models on the CC Energies of the 10 000 Molecule Test Set

parameterization	MAE energy (kcal/mol)	MAE dipole (eÅ)	MAE charge (e)
Auorg	10.55	0.079	0.085
MIO	10.69	0.079	0.085
GFN1-xTB	10.66	0.136	0.103
GFN2-xTB	13.03	0.153	0.089
Repulsive CC 20000	5.41	0.079	0.085
DFTBML CC 20000	2.67	0.033	0.053
DFTBML CC 2500	2.95	0.036	0.054
DFTBML CC 300	4.14	0.052	0.066

For the energy, the training and test errors converge at about 20 000 configurations, indicating that the training is saturated and additional data is unlikely to improve performance. For dipoles and charges, the training, validation, and testing losses track each other closely. This likely reflects the high weighting of energy in the loss function such that specialization of the model to the training data occurs only for the energy. It is a bit unusual that for dipoles and charges, the test error is smaller than the train error. However, this behavior inverts if the training and test sets are switched, suggesting that the training set has configurations somewhat more difficult than those of the test set.

DFTBML substantially improves upon the standard DFTB parametrizations, Auorg^40^ and MIO,^37^ as well as both GFN1-xTB and GFN2-xTB^52−55^ (Table 2). Auorg is a more direct comparison than MIO as both Auorg and DFTBML are shell-resolved, where Coulombic interactions differ between atomic shells (e.g., 2s versus 2p). Compared to Auorg, DFTBML CC 20000 gives a percent improvement of approximately 75% for total energy, 58% for dipole, and 38% for atomic charges. The improvement is largest for total energy, which is consistent with a greater emphasis being placed on total energy in the loss function. Comparison with “Repulsive 20000” in Table 2 indicates that only half of the improvement arises from the short-range repulsive potential, emphasizing the benefits of training both the electronic and repulsive components. Similar results are observed when fitting to the DFT total energy target (see Section S13), suggesting that the performance of DFTBML is not strongly dependent on the level of *ab initio* theory used to generate the target quantities.

The results of Table 2 are on the standard test set of 10 000 molecules. For the full ANI-1CCX data set, the performance of 2.90 kcal/mol for “DFTBML CC 20000” is comparable to that of 3.19 kcal/mol for DFT wB97x/def2-TZVPP in Figure 1. Examination of the orbital energies and interaction functions confirms that the parameters are physically reasonable (Section S9). This suggests that SKF-DFTB is a sufficiently flexible model form that, when trained to *ab initio* data, the resulting model has accuracy comparable to that of commonly used high-cost methods such as DFT.

We next consider two experiments that help reveal the extent to which DFTBML is learning the physics of the interactions present in these systems. The first is the far-transfer experiments discussed above, where the model is trained on 2500 configurations with up to five heavy atoms and tested on molecules with 6–8 heavy atoms (Table 3). Because the functions being learned by DFTBML go to zero beyond 4.5 Å and such distances are present in molecules with up to five heavy atoms, we may expect the performance in far-transfer experiments to be close to that of near-transfer. For far-transfer, DFTBML improves on Auorg by 59% for energy, 43% for dipole, and 26% for charges. For near-transfer with 2500 training configurations, the analogous improvements are 72% for energy, 54% for the dipole, and 36% for charges. These results suggest that DFTBML can learn from molecules with only up to five heavy atoms, as expected based on the range of the interactions being learned. The somewhat better performance seen in near-transfer may reflect the greater chemical diversity present in molecules with up to eight heavy atoms.

**Table 3:** Performance of Various Models on the Test Data Used for Far-Transfer Experiments, Where DFTBML is Trained on Molecules with up to Five Heavy Atoms and Tested on Molecules with 6–8 Heavy Atoms

parameterization	MAE energy (kcal/mol)	MAE dipole (eÅ)	MAE charge (e)
Auorg CC	11.81	0.089	0.088
MIO CC	11.86	0.089	0.088
Auorg DFT	13.25	0.089	0.088
MIO DFT	13.05	0.089	0.088
GFN1-xTB CC	10.85	0.147	0.104
GFN2-xTB CC	13.51	0.165	0.091
GFN1-xTB DFT	10.14	0.147	0.104
GFN2-xTB DFT	12.26	0.165	0.091
Transfer CC 2500	4.82	0.051	0.065
Transfer DFT 2500	4.79	0.050	0.063

**Figure 7:**
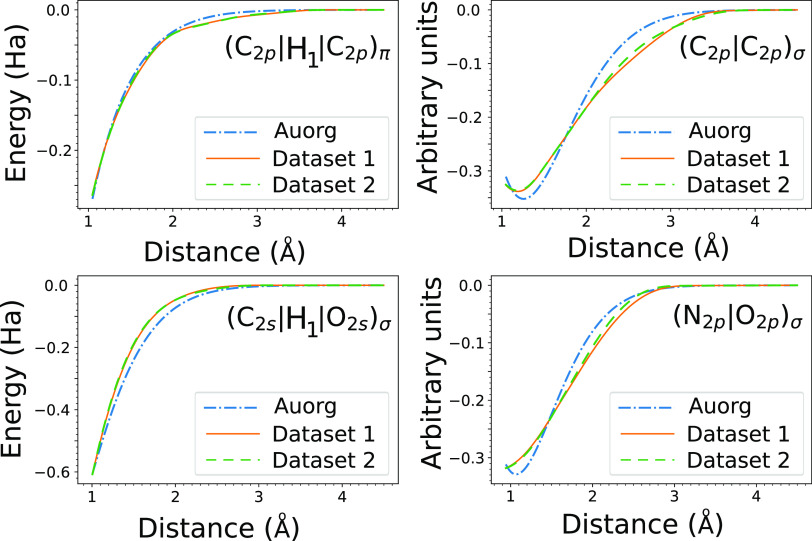
Example splines for Hamiltonian elements (**H**_**1**_, left) and overlap elements (**S**, right) generated from DFTBML training on the CC targets of disjoint data sets, each containing 5000 molecules. The Auorg potentials are included for reference.

A second experiment that explores the extent to which DFTBML is learning the underlying physics examines the sensitivity of the model parameters to training data. For this, we train to two nonoverlapping sets of training molecules, obtained by splitting the data set “DFTBML CC 10000” into two halves. The performance of the resulting models is in close agreement on all targets (Table 4), as are the resulting model forms (see Figure 7). That the resulting models are not sensitive to the specific data used to train the model suggests that DFTBML is learning the underlying physical interactions.

**Table 4:** Performance of DFTBML Trained on Two Disjoint Training Sets

parameterization	MAE energy (kcal/mol)	MAE dipole (eÅ)	MAE charge (e)
Auorg	10.55	0.079	0.085
MIO	10.69	0.079	0.085
GFN1-xTB	10.66	0.136	0.103
GFN2-xTB	13.03	0.153	0.089
DFTBML CC 5000 first half	2.83	0.037	0.058
DFTBML CC 5000 second half	2.90	0.038	0.051

### 2.5 COMP6 Benchmark Performance

To explore transfer of the model to molecules well outside the above training and testing data, we report the performance of DFTBML on the COMP6 benchmark suite developed by Isayev and colleagues.^56^ The benchmark suite contains a series of chemically diverse molecules, including an expansion on the S66x8 benchmark, frames obtained from molecular dynamics using the ANI-1x potential, subsets of molecules with various numbers of heavy atoms, and pharmacologically relevant structures (Table 5).

**Table 5:** Details on the DFTBML Parametrizations (First Three Rows) and the COMP6 Benchmarks (Remaining Rows) on Which These are Tested

parameterization/COMP6 set	description
DFTBML CC/DFT	trained on 20 000 molecules, 1–8 heavy atoms
Repulsive CC/DFT	trained on 20 000 molecules, 1–8 heavy atoms
Transfer CC/DFT	trained on 2500 molecules, 1–5 heavy atoms
Ani MD	1791 molecules with 11–158 heavy atoms
drugbank	13 379 molecules with 3–65 heavy atoms
GDB 7–13	83 670 molecules total; each GDB *n* contains molecules with *n* heavy atoms
S66x8	528 molecules with 2–16 heavy atoms
tripeptide	1979 molecules with 17–37 heavy atoms

The results are presented for Auorg and MIO, GFN1-xTB, and GFN2-xTB, along with several of the DFTBML parameter sets discussed above. To compare atomization energies, a linear reference energy term is refitted for each comparison (see eq 2 in Section 4). Tables 6–8 show the performance for energy, dipoles, and charges, respectively. Energies are reported per atom to aid comparisons across test sets that contain molecules with vastly different sizes, and to allow comparison to published results on HIPNN+SEQM,^36^ an alternative approach to semiempirical machine learning that uses neural networks.

**Table 6:** MAE of Total Energy in eV/Atom for DFTBML and Various Models on the COMP6 Benchmark Suite^a^

parameterization	Ani MD	GDB	drugbank	S66x8	tripeptide
Auorg	0.0056	0.0258	0.0151	0.0121	0.0078
MIO	0.0050	0.0252	0.0143	0.0100	0.0077
GFN1-xTB	0.0051	0.0216	0.0124	0.0116	0.0052
GFN2-xTB	0.0092	0.0214	0.0122	0.0118	0.0062
DFTBML CC	0.0048	0.0112	0.0086	**0.0071**	0.0043
Repulsive CC	0.0064	0.0170	0.0120	0.0128	0.0066
DFTBML DFT	0.0040	0.0082	**0.0070**	0.0072	**0.0034**
Repulsive DFT	0.0053	0.0151	0.0107	0.0124	0.0052
Transfer CC	0.0042	0.0126	0.0094	0.0101	0.0052
Transfer DFT	**0.0032**	0.0089	0.0072	0.0077	0.0041
HIPNN+SEQM	0.0110	**0.0070**	0.0090	0.0140	0.0070

a The lowest MAEs for each column are in bold. Numbers for HIPNN+SEQM are from Zhou et al.36

**Table 7:** MAE of Dipole in eÅ for DFTBML and Various Models on the COMP6 Benchmark Suite^a^

parameterization	Ani MD	GDB	drugbank	S66x8	tripeptide
Auorg	0.167	0.105	0.113	0.062	0.128
MIO	0.168	0.105	0.113	0.062	0.128
GFN1-xTB	0.170	0.161	0.208	0.129	0.311
GFN2-xTB	0.205	0.182	0.243	0.146	0.371
DFTBML CC	**0.104**	**0.040**	**0.053**	**0.031**	0.073
Repulsive CC	0.167	0.105	0.113	0.062	0.128
DFTBML DFT	0.106	0.042	0.055	0.033	**0.070**
Repulsive DFT	0.167	0.105	0.113	0.062	0.128
Transfer CC	0.119	0.057	0.066	0.043	0.079
Transfer DFT	0.115	0.054	0.063	0.040	0.074

a The lowest MAEs for each column are in bold.

**Table 8:** MAE of Charge in e for DFTBML and Various Models on the COMP6 Benchmark Suite^a^

parameterization	Ani MD	GDB	drugbank	S66x8	tripeptide
Auorg	0.071	0.071	0.065	0.065	0.085
MIO	0.071	0.071	0.065	0.065	0.085
GFN1-xTB	0.090	0.096	0.090	0.094	0.100
GFN2-xTB	0.076	0.084	0.074	0.084	0.087
DFTBML CC	**0.053**	**0.047**	**0.048**	**0.045**	**0.056**
Repulsive CC	0.071	0.071	0.065	0.065	0.085
DFTBML DFT	0.057	0.051	0.052	0.048	0.060
Repulsive DFT	0.071	0.071	0.065	0.065	0.085
Transfer CC	0.058	0.054	0.052	0.049	0.064
Transfer DFT	0.057	0.053	0.051	0.047	0.062

a The lowest MAEs for each column are in bold.

Molecules that are outliers or for which self-consistent field (SCF) iterations failed to converge are excluded from the comparisons. Such situations were rare with DFTBML having no outliers and three convergence failures for the GDB test suite. xTB had one outlier for GDB and a few hundred convergence failures for the Ani MD and drugbank test suites (see Table S16).

From the results shown in Tables 6–8, DFTBML models performed the best in every case except for the GDB test set, where HIPNN+SEQM performs slightly better. For total molecular energy, DFTBML performed better when trained against DFT data than CC data, which is not surprising given that the energies in the COMP6 data sets are from DFT with the wB97x functional and the 6-31G(d) basis set. For dipoles and charges, the DFTBML model trained to CC energies performs somewhat better than that trained to DFT energies (Tables 7 and 8). This is somewhat surprising given that in the CC training data, the dipoles and charges are from DFT.

In Table 6, the DFTBML parameters for “Transfer DFT/CC” were trained on 2500 molecules with up to five heavy atoms, while the parameters for “DFTBML DFT/CC” were trained on 20 000 molecules with up to eight heavy atoms. Comparison of the results shows that training to a larger set of data does tend to improve performance on the COMP6 tests but the improvements are modest. This further illustrates that reasonable DFTBML models can be obtained with relatively small amounts of training data.

Although the DFTBML model is not trained to forces, by using DFTB+ to compute the forces from the trained parameters, we can examine the degree to which the training of the model improves the performance on forces. Forces are available only for DFT in ANI-1CCX. For our standard test set of 10 000 molecules, the MAE in forces is improved by 40%, relative to standard DFTB parameters, i.e., comparing predictions of “DFTBML DFT 20000” to Auorg. This is less than the 76% improvement seen in energy (for which MAE drops from 11.95 kcal/mol for Auorg to 2.84 kcal/mol for “DFTBML DFT 20000” in Table S29). This suggests that extending the approach to allow for explicit training to forces would be a worthwhile extension. More information can be found in Section S14.

## 3 Conclusions

Here, we develop and evaluate a semiempirical quantum chemical model that can learn from large data sets while maintaining a physics-based and interpretable form. The resulting DFTBML model reduces the prediction error on the ANI-1CCX data set, relative to standard DFTB parametrizations, by up to 75% for energy, 58% for dipoles, and 38% for atomic charges. The model also transfers well to the COMP6 benchmark suite, with DFTBML improving substantially on standard DFTB parametrizations and outperforming both GFN1-xTB and GFN2-xTB. The performance of DFTBML is also somewhat better than HIPNN+SEQM^36^ on all but one of the COMP6 benchmarks. HIPNN+SEQM is similar to DFTBML in that the approach uses data to improve the parameters of a semiempirical Hamiltonian. HIPNN+SEQM uses a neural network to make a subset of the parameters in the PM3 Hamiltonian functions of the environment of the atom. The neural networks provide the flexibility needed for the model to learn from training data; however, the neural networks function as black boxes, which are difficult to interpret. Here, the ability of the semiempirical model to learn from data is imparted by the use of a flexible form for the one-dimensional functions that describe the dependence of the interactions on the interatomic distance. Regularizations are applied to ensure these functions have reasonable physical forms such that the model is physics-based and interpretable. As a result, DFTBML is able to learn from the data and achieve a performance equivalent to that of HIPNN+SEQM while maintaining an interpretable form.

The interpretability of the DFTBML model has two related aspects. The first is emphasized in this work, that the Hamiltonian and model parameters can be examined to understand the physics that is included and excluded from the model predictions. The other aspect relates to the intermediate quantities, such as orbital energies and populations, that come from a physics-based model. Chemists often use this additional information to make sense of the results they obtain from quantum chemical calculations^57^ and gain insights that go beyond numerical predictions for energy, dipole, and other specific targets.

An alternative approach to integrating ML into the DFTB model form has been explored by Fan et al.^58,59^ DFTB obtains the electronic parameters from DFT solutions for isolated atoms that are placed in a confinement potential to include effects from the surrounding atoms. The approach explored by Fan et al. uses ML to make the confinement potential a function of the atomic environment. This helps ensure that the energies, interactions, and overlaps of the DFTB Hamiltonian are consistent in that they can be traced back to atomic orbitals. This approach has been shown to improve the accuracy of DFTB for charges, dipoles, and charge population analyses on molecules with up to five heavy atoms. The improvement in accuracy for dipole moments is comparable to that of the DFTBML models reported here.

In addition to providing an interpretable model, the data requirements for DFTBML are substantially smaller than the millions of molecules needed for deep learning. Reasonable results are obtained when DFTBML is trained to as few as 300 molecules and the training saturates at about 20 000 molecules. Given that generation of the *ab initio* training data is a main computational bottleneck in model development, this reduction in required training data is a substantial practical advantage over deep learning. The saturation of DFTBML observed with greater amounts of training data also suggests that the performances reported here reflect the limit of a SKF-DFTB model, and that further enhancements in accuracy may require improvements to the model Hamiltonian itself, or the use of context-sensitive parameters such as in HIPNN+SEQM.^36^ Such extensions to the model may also help with training to multiple targets, relaxing the current trade-off between the accuracy of energy and dipole targets (see Figure 5).

The work here is restricted to SCF solutions for distorted structures of organic molecules consisting of C, N, O, and H. Future work includes extensions to additional elements such as transition metals, additional properties^60^ such as excitation energies^61,62^ and reaction barriers,^63,64^ and inclusion of additional interactions such as dispersion^65,66^ and solvent interactions.^67,68^

## 4 Experimental Details

**Figure 8:**
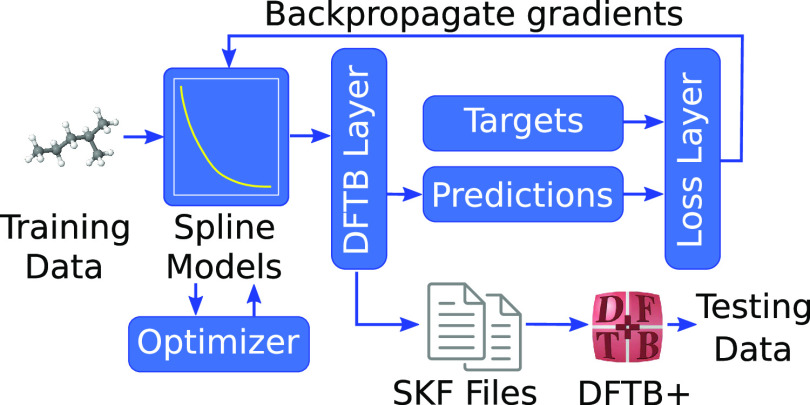
High-level overview of the DFTBML model workflow. Note that model testing uses DFTB+ and is external to model training (lower right).

Figure 8 gives an overview of the computational workflow used in training the DFTBML model within PyTorch.^41^ The DFTB layer^44^ is central to the DFTBML model as it solves the quantum chemical system for the desired properties on each forward pass. The training and validation data are randomly divided into batches, which each contain 10 configurations. During each epoch of the training process, the batches are randomly shuffled and fed through the model. Since a training experiment can consist of thousands of epochs, a precomputation is used to calculate and save quantities that do not depend on trained model parameters. This significantly decreases the training time but does come at the cost of increased memory usage and fixed batch compositions. The results presented here use a PyTorch implementation that is much more flexible than the previous TensorFlow implementation.^44^ In particular, batches are no longer required to have the same sequence of empirical formulas.

**Figure 9:**
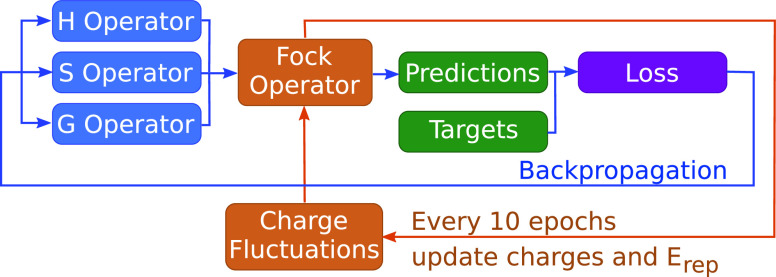
Schematic illustration of inverting the SCF (orange arrows) and training (blue arrows) loops of the DFTBML workflow. In the outer loop, the charge fluctuations needed for the Fock operator are updated based on the current model parameters. The repulsive model is updated on the same schedule as for the charge fluctuations.

To enable efficient backpropagation through SCF calculations during training, the SCF and training loops are inverted, as shown in Figure 9. This loop inversion scheme avoids backpropagation through multiple SCF cycles and instead moves the update of the charge fluctuations, required for the construction of the Fock operator, outside of the gradient descent steps used to improve the model parameters.^44^ SCF calculations are performed every 10 epochs throughout training. Updates to the repulsive model are also done every 10 epochs. Because the repulsive model and associated regularizations are linear, convex optimization can be used to find the global optimum for the entire training set. Implementation details of the repulsive model can be found in Section S4.

Comparisons between different quantum chemical methods are done with regard to atomization energies. This is implemented through a linear reference energy correction, which has the following form
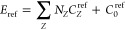
2where the sum is over elements, *N*_*Z*_ is the number of times element *Z* appears in the molecule, *C*_*Z*_^ref^ is a coefficient for element *Z*, and *C*_0_^ref^ is a constant term. The coefficients are obtained through a least-squares fit of *C*_*Z*_^ref^ and *C*_0_^ref^ in eq 2 to the energy differences between the two quantum chemical methods being compared. The reported MAEs refer to the residuals from this least-squares fit. Past work has found inclusion of *C*_0_^ref^ to have a small, but nonnegligible, effect on the results.^44^ Note that, because the reference energy has no impact on energy differences between chemical systems that contain the same number and types of elements, this approach to comparing methods is appropriate for evaluating accuracy on physically observable energies.

While training DFTBML, the reference energy is incorporated into the repulsive potential (see Section S4) such that the parameters of eq 2 are optimized during training. In this manner, the energy component of the loss function is based on a comparison of predicted and target atomization energies.

All experiments presented use the ADAM optimizer with a learning rate of 1 × 10^–05^ and the default values for all other parameters.^69^ All models were trained for 2500 epochs, with a learning rate scheduler that reduces the learning rate by a factor of 0.9 when a plateau is detected in performance improvements. The loss function combines the root-mean-square error for multiple targets, with weights of 6270 Ha^–1^ for total energy (Ha), 100 (eÅ)^−1^ for dipoles (eÅ), and 1 e^–1^ for charges (e). The sensitivity of the results to the number of knots in the splines and weights for the regularization penalties are provided in Section S12. The results for xTB use the standard implementations of GFN1-xTB and GFN2-xTB^52−55^ distributed via Anaconda. In reporting model performance, outliers are removed based on a threshold of 20 standard deviations above the mean error for total energy. For the standard test set used in training and testing DFTBML, there were less than five outliers when trained to 5000 or fewer molecules, and no outliers when trained to 10 000 or 20 000 molecules (see Tables S28 and S29). To facilitate comparison to other studies, including HIPNN+SEQM,^36^ per atom and per heavy atom results are provided in the Supporting Information.

## Data Availability

The code and data used to generate these results can be downloaded from the GitHub repository at https://github.com/djyaron/DFTBML/.

## Supporting Information Available

Additional information on the implementation of the DFTBML model, computer timings, hyperparameter tunings, and results (PDF)
