# Supplementary material for: Treating Semiempirical Hamiltonians as Flexible Machine Learning Models Yields Accurate and Interpretable Results
Source: J Chem Theory Comput. 2023 Sep 14;19(18):6185–96. doi: 10.1021/acs.jctc.3c00491 (PMC10536991; doi:10.1021/acs.jctc.3c00491)
Supplement: Supplementary file 1 — ct3c00491_si_001.pdf [file ct3c00491_si_001.pdf]

Supporting Information: Treating Semiempirical Hamiltonians as Flexible Machine Learning Models yields Accurate and Interpretable Results

Frank Hu, Francis He, and David J. Yaron*

Department of Chemistry, Carnegie Mellon University, Pittsburgh, PA

E-mail: yaron@cmu.edu

Contents

**S1**	**DFTBML model timing**	**S-2**
**S2**	**Spline Implementation**	**S-5**
**S3**	**The DFTB method**	**S-7**
**S4**	**Repulsive model formulation**	**S-9**
**S5**	**Parameterization of the repulsive potential in SKF-DFTB**	**S-12**
**S6**	**Spline regularization**	**S-13**
**S7**	**Backpropagation and degeneracy**	**S-16**
**S8**	**Tabulated spline cutoffs**	**S-18**

S9 Atomic energies and Hubbard parameters **S-20**

S10 Detailed dataset generation scheme **S-23**

S11 Outlier exclusion method **S-26**

S12 Hyperparameter investigation **S-28**

S12.1 The effect of the charge and dipole weighting factors S-29

S12.2 The effect of the spline knot sequence S-32

S12.3 The effect of the inflection point position S-34

S12.4 The effect of the third derivative penalty weight S-36

S13 Tables and figures for Section 2 **S-39**

S13.1 A note on learning curves S-39

S13.2 Repulsive potential performance S-39

S13.3 Unregularized model performance S-44

S13.4 Model performance when training on different dataset sizes S-45

S13.5 Model transferability and reproducibility S-50

S13.6 DFTBML performance on COMP6 benchmark S-52

S14 Analysis of forces **S-53**

References **S-56**

S1 DFTBML model timing

For reporting the DFTBML timings, the total process time in hours is used which is the sum of the total system and user CPU time. A distinction is made between SCF and non-SCF epoch timings, since the SCF cycle, which is performed every 10 epochs, is done outside of the PyTorch code used to update parameters. Table S1 gives the average epoch time for both

SCF and non-SCF epochs (μ_{SCF} and $\mu_{non-SCF}$) and the standard deviation of the epoch times for SCF and non-SCF epochs (σ_{SCF} and $\sigma_{non-SCF}$). The percentage of the total time spent performing SCF calculations ($\%_{SCF}$) is also reported and is approximated as:

$$\%_{SCF} \approx \left(\frac{(\mu_{SCF} - \mu_{non-SCF}) * 250}{T_{tot}} \right) * 100 \tag{S1}$$

Where T_{tot} is the total amount of time taken for the experiment. The factor of 250 is used because each experiment is run for 2500 epochs and charge updates happen every 10 epochs, meaning a total of 250 epochs where there is an additional SCF time added on top of the normal epoch runtime. This quantity is not the exact breakdown but serves to give an idea of how much time is spent in the SCF cycle relative to the rest of the training process. The repulsive update calculation that happens every 10 epochs is not included in the measured timings since solving the convex optimization problem takes negligible time relative to charge updates even when training on 20000 molecules. All experiments were run using 16 CPU cores.

Table S1: Total CPU process time used for different experiments in hours

**Parameterization**	$\mu_{non-SCF}$	$\sigma_{non-SCF}$	μ_{SCF}	σ_{SCF}	T_{tot}	$\%_{SCF}$
DFTBML 20000 CC	0.81	0.1034	1.81	0.2385	2279.40	10.89
DFTBML 10000 CC	0.49	0.0169	1.13	0.0505	1380.48	11.53
DFTBML 5000 CC	0.24	0.0089	0.53	0.0213	676.49	10.84
DFTBML 2500 CC	0.12	0.0040	0.27	0.0085	338.08	11.11
DFTBML 1000 CC	0.05	0.0030	0.13	0.0075	150.25	12.51
DFTBML 300 CC	0.02	0.0007	0.07	0.0020	68.24	16.38
DFTBML 20000 DFT	0.88	0.1217	1.94	0.2741	2455.31	10.79
DFTBML 10000 DFT	0.37	0.0066	0.83	0.0166	1046.61	10.90
DFTBML 5000 DFT	0.24	0.0086	0.54	0.0169	680.12	10.90
DFTBML 2500 DFT	0.12	0.0033	0.27	0.0078	338.15	11.32
DFTBML 1000 DFT	0.05	0.0021	0.13	0.0055	150.08	12.46
DFTBML 300 DFT	0.02	0.0006	0.07	0.0014	69.20	15.96

From Table S1, it is evident that the amount of time taken scales roughly linearly with the amount of data used, where doubling the data approximately doubles T_{tot}. There is

also no systematic difference between the timing performance when training to the CC total energy target versus the DFT total energy target. Furthermore, the percentage of time spent performing the required SCF calculations takes up less than 20% for all experiments, and $\%_{SCF}$ increases as the total amount of training time decreases, which is expected.

S2 Spline Implementation

For a spline in a B-spline basis of order k, a prediction y for a given input x is generated through the linear combination of the set of N B-spline basis functions $\{b_j^k\}$ using the set of coefficients $\{\beta_j\}$ as follows:

$$y = \sum_{i=1}^{N} \beta_i b_i^k(x) + \beta_0 \tag{S2}$$

For DFTBML, the input x corresponds to an interatomic distance. Instead of predicting a single value at a time, we now wish to predict a vector of values $\mathbf{y}$, essentially performing the following transformation:

$$y_j = \sum_{i=0}^{N} \beta_i b_i^k(x_j), \forall j \tag{S3}$$

Where the constant term β_0 is subsumed into the summation and $b_0^k = 1$. Now, we can transform this into matrix form by introducing a second index j as follows:

$$y_j = \sum_{i=0}^{N} \beta_i b_{i,j}^k, \forall j \tag{S4}$$

$$\mathbf{y} = (b_{j,i}^k)\boldsymbol{\beta} \tag{S5}$$

The quantity $(b_{j,i}^k)$ is the spline basis matrix, and $\boldsymbol{\beta}$ is the coefficient vector. Setting $\mathbf{A} = (b_{j,i}^k)$ and $\mathbf{x} = \boldsymbol{\beta}$, we get the matrix multiplication

$$\mathbf{y} = \mathbf{A}\mathbf{x} + \mathbf{c} \tag{S6}$$

Where $\mathbf{c}$ is used to account for boundary conditions specifying fixed values.

Predictions for higher derivatives can be obtained for the spline models with the same coefficient vector $\mathbf{x}$ for each derivative. From Equation S4, predicting the value of a higher

derivative can be done as follows:

$$y_j^{(n)} = \frac{d^n}{dx^n} \sum_{i=0}^{N} \beta_i b_{i,j}^k = \sum_{i=0}^{N} \beta_i b_{i,j}^{(n),k}, \forall j \tag{S7}$$

$$\mathbf{y}^{(n)} = (b_{j,i}^{(n),k})\boldsymbol{\beta} \tag{S8}$$

Where n is the order of the derivative to calculate. Setting the matrix $\mathbf{A}^{(n)} = (b_{j,i}^{(n),k})$ gives a similar result to Equation S6, where:

$$\mathbf{y}^{(n)} = \mathbf{A}^{(n)}\mathbf{x} + \mathbf{c}^{(n)} \tag{S9}$$

S3 The DFTB method

A derivation of the DFTB method can be found in work by Elstner et al.[S1,S2] and in previous work by Li et al.[S3] For DFTBML, we include a classical pairwise repulsive term as well as a reference energy correction which takes the following form:

$$E_{ref} = \sum_{z\in\{m\}} N_z C_z^{ref} + C_0^{ref} \tag{S10}$$

Where $\{m\}$ is the set of all atomic numbers needed to describe a given molecule, N_z is the number of times atom z appears in the molecule, C_z^{ref} is the coefficient for atom z, and C_0^{ref} is a constant term. The coefficients are obtained through a least squares fit. In comparing two different quantum chemical methods, disagreements refer to to the residuals from this least-squares fit.

The parameters of DFTB are the Hubbard parameters that model the coulombic interactions within electron shells, on-site energies for each type of atomic orbital (e.g. 2s on C, 2p on N), neutral orbital occupations for each element, and the Hamiltonian and overlap integrals which form the Hamiltonian and overlap operator matrices.

Formally, the Hamiltonian integrals can be written as:

$$\left\langle \phi_\mu(\mathbf{r}) \middle| -\frac{1}{2}\nabla^2 + \nu_{\text{eff}}[n^\alpha(\mathbf{r})] + \nu_{\text{eff}}[n^\beta(\mathbf{r}-\mathbf{r_0})] \middle| \phi_\nu(\mathbf{r}-\mathbf{r_0}) \right\rangle, \mu \in \alpha, \nu \in \beta \tag{S11}$$

$$\left\langle \phi_\mu(\mathbf{r}) \middle| -\frac{1}{2}\nabla^2 + \nu_{\text{eff}}[n^\alpha(\mathbf{r}) + n^\beta(\mathbf{r}-\mathbf{r_0})] \middle| \phi_\nu(\mathbf{r}-\mathbf{r_0}) \right\rangle, \mu \in \alpha, \nu \in \beta \tag{S12}$$

Where the first case is the potential case and the second case is the superposition case. n^α and n^β are the atomic densities and ν_{eff} is the effective potential. ϕ_μ and ϕ_ν are the atomic valence basis functions for atom α and β, respectively, and $\mathbf{r}$ is the internuclear distance vector.

The overlap integrals can be written as:

$$\Big\langle \phi_\mu(\mathbf{r}) \Big| \phi_\nu(\mathbf{r} - \mathbf{r_0}) \Big\rangle, \mu \in \alpha, \nu \in \beta \tag{S13}$$

For both the Hamiltonian and overlap integrals, the integrals are evaluated in orientations corresponding to σ, π, and, for d orbitals, δ.

In traditional DFTB, approximate atomic orbitals are used to explicitly evaluate the integrals of Eqs. S11 through S13. In DFTBML, these integrals are instead derived from fits to the training data.

S4 Repulsive model formulation

The DFTB layer[S3] handles calculations of the electronic energy. However, the total molecular energy is comprised of the electronic (E_{elec}), repulsive (E_{rep}), and reference (E_{ref}) energies, i.e. $E_{tot} = E_{elec} + E_{rep} + E_{ref}$. Here, we provide an overview of the repulsive model which accounts for the E_{rep} and E_{ref} contributions.

The repulsive energy is modeled using pairwise additive models. Consider a set of molecules where each molecule contains D atoms with M many atom types. Then, the repulsive energy of a single molecule is:

$$E_{rep_{mol}} = \sum_{i<j}^{D} f_{Z_i,Z_j}(|r_i - r_j|), Z_i, Z_j \in M \tag{S14}$$

Where f_{Z_i,Z_j} denotes the pairwise function describing a repulsive interaction between atoms Z_i and Z_j that only depends on their internuclear distance $|r_i - r_j|$. In the DFTBML implementation, the set of functions $\{f_{Z_i,Z_j}\}$ is modeled using cubic splines represented in a B-spline basis. Rather than training the reference energy separately, it is incorporated into the repulsive energy so that the full formulation is as follows:

$$E_{newrep_{mol}} = \sum_{i<j}^{D} f_{Z_i,Z_j}(|r_i - r_j|) + \sum_{z}^{M} N_z C_z^{ref} + C_0^{ref} \tag{S15}$$

The first term in Equation S15 can be expressed as a matrix multiplication between a matrix that depends on the distances present in the molecule, and a vector that contains the trainable parameters associated with the spline, $\mathbf{x}$ and $\mathbf{c}$ of Equation S6. The relation between the energy and the trainable parameters in Eq. S15 is linear and so may, for each molecule, be written as,

$$E_{rep+ref}^{mol} = \boldsymbol{\gamma}_{mol}\mathbf{x} \tag{S16}$$

where $\mathbf{x}$ is a vector holding all training parameters and $\boldsymbol{\gamma}$ describes the linear relation between the energy of the molecule and these parameters.

Unlike the computation of the electronic energy through the DFTB layer, the repulsive energy is linear in model parameters and optimization does not require a gradient descent procedure. We instead use a quadratic programming approach[S4] to solve for the globally optimal solution, using the CVXOPT package in Python. Quadratic programming aims to solve the following program for the coefficient vector $\mathbf{x}$:

$$\underset{\mathbf{x}\in\{\mathbf{R}^n\}}{\operatorname{argmin}} \left(\frac{1}{2}\right) \mathbf{x}^T \mathbf{P} \mathbf{x} + \mathbf{q}^T \mathbf{x} \tag{S17}$$

$$\mathbf{G}\mathbf{x} \preceq \mathbf{h} \tag{S18}$$

$$\mathbf{A}\mathbf{x} = \mathbf{b} \tag{S19}$$

Where Equation S17 is linear least squares optimization, Equation S18 specifies an inequality constraint on the coefficient vector $\mathbf{x}$ and Equation S19 specifies an equality constraint. For repulsive potentials, the spline model is regularized by forcing the function to be monotonically decaying. This constraint, that the first derivative be negative, can be written as the linear inequality of Equation S18. For our application here, we do not apply the equality constraint of Equation S19.

The matrices $\mathbf{P}$ and $\mathbf{q}$ are:

$$\mathbf{P} = \sum_{mol} \boldsymbol{\gamma}_{mol}^T \boldsymbol{\gamma}_{mol} \tag{S20}$$

$$\mathbf{q} = -\sum_{mol} \left(\frac{1}{N_{mol}}\right) \mathbf{y}_{mol}^T \boldsymbol{\gamma}_{mol} \tag{S21}$$

Where the sum is over all molecular configurations, and $\mathbf{y}_{mol}$ is the target that the repulsive energy is being trained to. The target for the repulsive potential is the difference between the total molecular energy and the electronic energy. Both the predicted and target molecular

energies are divided by the number of heavy atoms, so that the optimization is performed on the energy per heavy atom.

S5 Parameterization of the repulsive potential in SKF-DFTB

The repulsive interaction is modeled as a cubic spline with a fifth degree polynomial to describe the final interval. For distances below where the spline begins, the repulsive energy is assumed to have the following form near the repulsive wall:

$$e^{-a_1 r + a_2} + a_3 \tag{S22}$$

Where a_1, a_2, and a_3 are constants specified in the file. In DFTBML, we do not train these constants and instead use the spline repulsive to describe all relevant repulsive interactions, thus spanning a range which encompasses all physically relevant distances.

The remaining form of the spline repulsive is a series of coefficients c_0, c_1, c_2, and c_3 which specify the following cubic function spanning the distance of the associated interval:

$$c_0 + c_1(r - r_0) + c_2(r - r_0)^2 + c_3(r - r_0)^3 \tag{S23}$$

The final series of coefficients specifies a fifth order polynomial with two additional coefficients c_4 and c_5:

$$c_0 + c_1(r - r_0) + c_2(r - r_0)^2 + c_3(r - r_0)^3 + c_4(r - r_0)^4 + c_5(r - r_0)^5 \tag{S24}$$

However, in DFTBML, this fifth order polynomial is reduced to a cubic one by setting $c_4 = c_5 = 0$ since the entirety of the repulsive potential is modeled using a cubic spline in a B-spline basis.

S6 Spline regularization

Formally, the loss for each batch takes on a Root-Mean-Square (RMS) definition as follows:

$$Loss = \sum_{prop} w_{prop} \sqrt{\frac{1}{N_{prop}} \sum_{i}^{N_{prop}} |Pred_i - Target_i|^2} + L_{form} \tag{S25}$$

Where the summation goes over all properties of interest and $Pred$ and $Target$ are the predicted and target values for each property, respectively. N_{prop} is the number of predicted values for each property, and w_{prop} is a weighting factor used to target certain attributes. The w_{prop} values are treated as hyperparameters throughout training. Multiple properties are considered in the loss function because DFTBML is a multitask learning model, where multiple targets are simultaneously optimized. Of interest here is total molecular energy, molecular dipole, and atomic charge.

The final term in Equation S25, L_{form}, is a regularization term for the electronic splines used to model elements of the Hamiltonian and overlap operator elements. L_{form} contains two terms, with one governing the curvature of the spline and the other penalizing the magnitude of the third derivative as follows:

$$L_{form} = w_{convex} * L_{convex} + w_{TD} * L_{TD} \tag{S26}$$

$$L_{convex} = \sqrt{\frac{1}{K} \sum_{mod}^{K} \frac{|ReLU(dsgn(mod)(\mathbf{y}_{mod}^{(2)} \circ \mathbf{p}_{mod}))|^2}{N_{mod}}} \tag{S27}$$

$$L_{TD} = \sqrt{\frac{1}{K} \sum_{mod}^{K} \sqrt{\frac{|\mathbf{y}_{mod}^{(3)}|^2}{N_{mod}}}} \tag{S28}$$

Where K is the number of spline models contained in the batch, $\mathbf{y}_{mod}^{(2)}$ is the vector of predicted second derivative values for the current indexed model, $\mathbf{y}_{mod}^{(3)}$ is the vector of predicted third derivative values for the current indexed model, and N_{mod} is the number of values predicted for the given model for the second or third derivatives. $ReLU$ is the

rectified linear unit function and $dsgn(\cdot)$ evalutes to 1 or -1 for the given model depending on the sign of the model's curvature. Because the spline models are univariate curvatures as a function of distance, each model is either mostly concave up or concave down. The $dsgn(\cdot)$ function returns 1 for a negative integral and -1 for a positive integral so that applying $ReLU$ selects the correct values to penalize.

For the overlap operator, there are cases where an inflection point can exist at short range. This inflection arises from the nodal structure of the atomic orbitals. For those cases where an inflection point is allowed, the penalty vector $\mathbf{p}_{mod}$ is multiplied element-wise into the predictions of the second derivative before the functions $dsgn(\cdot)$ and $ReLU$ are applied. The penalty vector $\mathbf{p}_{mod}$ is used to account for functional forms which have a change to upward curvature at short range, a phenomenon seen in the overlap integrals of the Auorg and MIO parameterizations. Figure S1 shows an example of this with the $\left(C_{2p}|C_{2p}\right)_\sigma$ overlap matrix element.

For the derivation of $\mathbf{p}_{mod}$, the first step is to define the functional form of the inflection point. In DFTBML, the inflection point is defined as follows:

$$r_{inflect} = r_l + \left(\frac{r_h - r_l}{2}\right)\left(\frac{2\arctan x}{\pi} + 1\right) \tag{S29}$$

Where r_l and r_h are the boundary values for a given model and x is the variable which is optimized during the gradient descent procedure. In this way, the inflection point is tied to a single variable which simplifies the training process. During training, the current value of the inflection point is calculated using Equation S29 and that value of $r_{inflect}$ is used to calculate $\mathbf{p}_{mod}$ as follows:

$$p_i = \arctan\left(10(r_i - r_{inflect})\right), \forall i \tag{S30}$$

Where the r_i are the distances corresponding to the predictions of the second derivative. This method means that for all distances in $\mathbf{p}_{mod}$ that are greater than the inflection point,

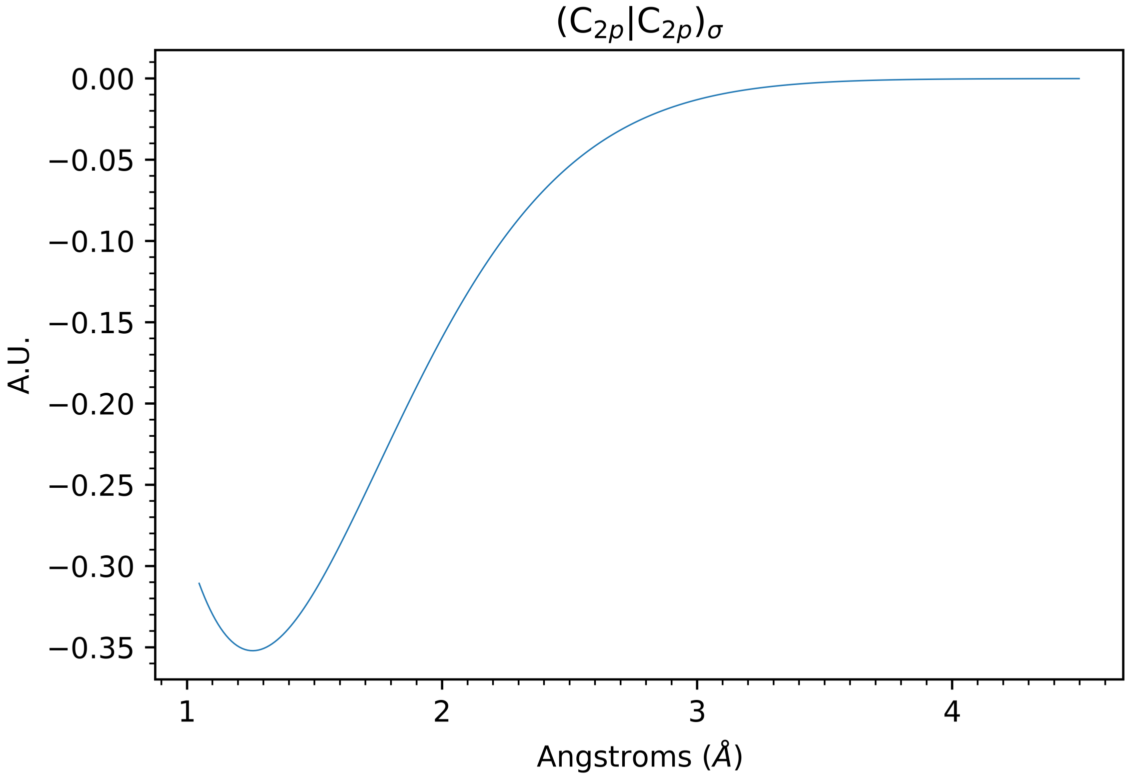

Figure S1: The spline used to model the overlap interaction between two carbon 2p orbitals in the σ orientation. The y-axis is represented in arbitrary units for the overlap integral.

you have a positive value whereas for all distances smaller than the inflection point, you have some negative value. Multiplying this into the second derivative vector allows for the sign of the second derivative to change once across the inflection point in a smooth and differentiable manner.

S7 Backpropagation and degeneracy

One of the key steps in the DFTB layer is the formation and diagonalization of the Fock matrix. In evaluating the gradients needed to backpropagate through the eigensystem, singularities arise for degenerate orbitals. These singularities are not related to the occupation of the orbitals, and arise even if the degenerate orbitals are fully occupied or are completely unoccupied. In the original work with the DFTB layer,[S3] the effects of these singularities were reduced by removing symmetric molecular configurations from the training data, with the symmetry being measured by the separation between orbital energies computed using the MIO DFTB parameters.[S1]

Here, we use a more general approach based on the eigenvalue broadening of Seeger et al.[S5] In the forward pass through the model, the symmetric eigendecomposition is as follows:

$$(\mathbf{U}, \boldsymbol{\lambda}) = syevd(\mathbf{A}) \tag{S31}$$

$$\mathbf{A} = \mathbf{U}^T(diag(\boldsymbol{\lambda}))\mathbf{U} \tag{S32}$$

$$\mathbf{U}^T\mathbf{U} = \mathbf{I} \tag{S33}$$

Where $syevd(\cdot)$ represents the symmetric eigenvalue decomposition function, $\mathbf{A}$ is the matrix we are decomposing, $\mathbf{U}$ is the matrix of eigenvectors, $\boldsymbol{\lambda}$ is the matrix of eigenvalues, and $\mathbf{I}$ is the identity matrix. Since $\mathbf{A}$ is specified as a symmetric matrix, the eigenvectors $\mathbf{U}$ are unitary, as seen in Equation S33.

The backward pass is performed as follows:

$$\bar{\mathbf{A}} = \mathbf{U}^T(sym(\bar{\mathbf{U}}\mathbf{U}^T \circ \mathbf{F}) + \bar{\boldsymbol{\Lambda}})\mathbf{U} \tag{S34}$$

$$F_{ij} = \frac{I_{\{i \neq j\}}}{h(\lambda_i - \lambda_j)} \tag{S35}$$

$$h(t) = max(|t|, \epsilon)sgn(t) \tag{S36}$$

Where $\mathbf{U}$ and $\boldsymbol{\lambda}$ are the outputs from the forward pass and $\bar{\mathbf{U}}$ are the gradients for the matrix

$\mathbf{U}$ and $\bar{\mathbf{\Lambda}}$ is the diagonal matrix formed from the gradients for the eigenvalues, $\bar{\boldsymbol{\lambda}}$. $sgn(\cdot)$ is the sign function which returns ± 1 depending on the sign of t. Equations S35 and S36 specify a conditional eigenvalue broadening behavior, whereby if an eigengap between two eigenvalues λ_i and λ_j, $i \neq j$, is smaller than a fixed constant ϵ, the value of ϵ is substituted instead. This represents a tradeoff between accuracy and stability, since the backward pass is now able to handle vanishing eigengaps but the results of the backward pass can only be treated as approximate rather than exact. For this study, we use a value of 1E-12 for ϵ, a small scalar which provides the numerical stability to process all systems of interest without overly compromising on accuracy.

S8 Tabulated spline cutoffs

This section presents the tabulated cutoffs r_h and r_l for both the electronic and repulsive splines. Values for these cutoffs were determined through a distribution analysis as explained in Section 4 of the main paper. It is important to note that when using the electronic potentials generated from DFTBML, the data that the potentials are applied to cannot contain configurations with internuclear distances lower then the r_l value of a given atom pair. This is equivalent to extrapolating beyond the trained region of the spline and can lead to unreasonable or incorrect results. Distances greater than r_h are allowed because the potentials converge to 0 past this distance.

Table S2: Low- and high-end cutoffs for the fifth-order splines used for the Hamiltonian ($\mathbf{H_1}$) and Overlap ($\mathbf{S}$) operators of DFTBML.

**Element pair**	$\mathbf{r_l}$ **(Å)**	$\mathbf{r_h}$ **(Å)**
H-H	0.5	4.5
C-C	1.04	4.5
H-C	0.602	4.5
N-N	0.986	4.5
C-N	0.948	4.5
H-N	0.573	4.5
H-O	0.599	4.5
C-O	1.005	4.5
N-O	0.933	4.5
O-O	1.062	4.5

Table S3: Low- and high-end cutoffs for the third-order splines used for the repulsive potentials ($\mathbf{R}$) of DFTBML.

**Element pair**	$\mathbf{r_l}$ **(Å)**	$\mathbf{r_h}$ **(Å)**
H-H	0	2.10
C-C	0	1.80
H-C	0	1.60
N-N	0	1.80
C-N	0	1.80
H-N	0	1.60
H-O	0	1.50
C-O	0	1.80
N-O	0	1.80
O-O	0	1.80

S9 Atomic energies and Hubbard parameters

Presented here are some tables for the atomic energies and Hubbard parameters, as well as the extend to which they changed, relative to those of Auorg, during training. Notation wise, E_s and E_p are the energies for the s and p orbitals respectively, and U_s and U_p are the Hubbard parameters for the s and p orbitals. The energies of the d orbitals (e.g. E_d and U_d) are excluded as they are zero for first- and second-row elements.

Table S4: Energies and Hubbard parameters for DFTBML CC 20000

**Element**	$\mathbf{E_p}$	$\mathbf{E_s}$	$\mathbf{U_p}$	$\mathbf{U_s}$
H	0.000	−0.215	0.000	0.368
C	−0.209	−0.497	0.399	0.420
N	−0.276	−0.670	0.443	0.673
O	−0.336	−0.902	0.532	0.696

Table S5: Energies and Hubbard parameters for DFTBML DFT 20000

**Element**	$\mathbf{E_p}$	$\mathbf{E_s}$	$\mathbf{U_p}$	$\mathbf{U_s}$
H	0.000	−0.209	0.000	0.362
C	−0.207	−0.495	0.378	0.391
N	−0.276	−0.663	0.440	0.634
O	−0.333	−0.901	0.533	0.733

Table S6: Energies and Hubbard parameters for DFTBML CC 2500

**Element**	$\mathbf{E_p}$	$\mathbf{E_s}$	$\mathbf{U_p}$	$\mathbf{U_s}$
H	0.000	−0.211	0.000	0.365
C	−0.204	−0.498	0.397	0.409
N	−0.274	−0.661	0.444	0.628
O	−0.334	−0.893	0.512	0.571

Table S7: Energies and Hubbard parameters for DFTBML DFT 2500

**Element**	$\mathbf{E_p}$	$\mathbf{E_s}$	$\mathbf{U_p}$	$\mathbf{U_s}$
H	0.000	−0.207	0.000	0.368
C	−0.201	−0.495	0.435	0.445
N	−0.275	−0.662	0.470	0.667
O	−0.334	−0.888	0.543	0.661

Table S8: Energies and Hubbard parameters for DFTBML CC 300

**Element**	$\mathbf{E_p}$	$\mathbf{E_s}$	$\mathbf{U_p}$	$\mathbf{U_s}$
H	0.000	−0.222	0.000	0.363
C	−0.193	−0.508	0.365	0.365
N	−0.272	−0.646	0.423	0.437
O	−0.333	−0.880	0.511	0.538

Table S9: Energies and Hubbard parameters for DFTBML DFT 300

**Element**	$\mathbf{E_p}$	$\mathbf{E_s}$	$\mathbf{U_p}$	$\mathbf{U_s}$
H	0.000	−0.227	0.000	0.374
C	−0.197	−0.508	0.380	0.376
N	−0.264	−0.644	0.387	0.500
O	−0.332	−0.879	0.484	0.527

Table S10: Changes in energies and Hubbard parameters for DFTBML CC 20000

**Element**	$\mathbf{E_p}$	$\mathbf{E_s}$	$\mathbf{U_p}$	$\mathbf{U_s}$
H	0.000	0.024	0.000	−0.051
C	−0.015	0.007	0.034	0.056
N	−0.015	−0.030	0.012	0.242
O	−0.004	−0.023	0.037	0.201

Table S11: Changes in energies and Hubbard parameters for DFTBML DFT 20000

**Element**	$\mathbf{E_p}$	$\mathbf{E_s}$	$\mathbf{U_p}$	$\mathbf{U_s}$
H	0.000	0.029	0.000	−0.058
C	−0.012	0.010	0.014	0.026
N	−0.015	−0.023	0.009	0.203
O	−0.001	−0.022	0.038	0.238

Table S12: Changes in energies and Hubbard parameters for DFTBML CC 2500

Element	E_p	E_s	U_p	U_s
H	0.000	0.027	0.000	−0.055
C	−0.010	0.007	0.033	0.044
N	−0.014	−0.021	0.013	0.197
O	−0.002	−0.014	0.017	0.076

Table S13: Changes in energies and Hubbard parameters for DFTBML DFT 2500

Element	E_p	E_s	U_p	U_s
H	0.000	0.031	0.000	−0.051
C	−0.007	0.009	0.070	0.080
N	−0.015	−0.022	0.039	0.236
O	−0.002	−0.009	0.048	0.166

Table S14: Changes in energies and Hubbard parameters for DFTBML CC 300

Element	E_p	E_s	U_p	U_s
H	0.000	0.017	0.000	−0.056
C	0.002	−0.004	0.000	0.000
N	−0.011	−0.006	−0.008	0.006
O	−0.001	−0.002	0.016	0.043

Table S15: Changes in energies and Hubbard parameters for DFTBML DFT 300

Element	E_p	E_s	U_p	U_s
H	0.000	0.012	0.000	−0.046
C	−0.002	−0.003	0.015	0.011
N	−0.004	−0.004	−0.043	0.069
O	0.000	0.000	−0.011	0.031

S10 Detailed dataset generation scheme

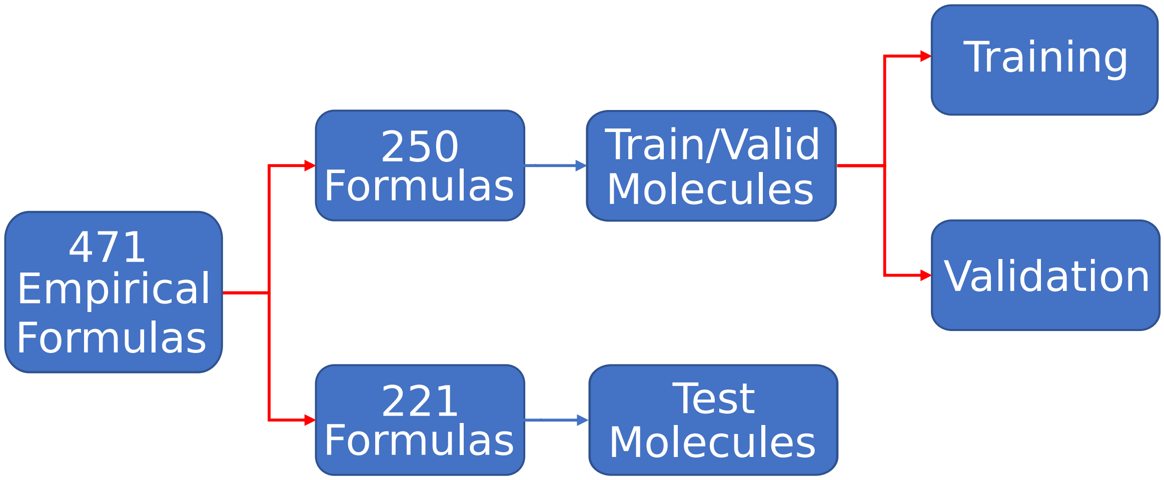

Figure S2: High-level overview of the method used to generate the initial dataset. Red arrows indicate random sampling.

For a more detailed explanation of the dataset generation scheme for the base dataset, the workflow consists of the following steps:

1. A subset of the 471 empirical formulas is randomly chosen to be used for the training and validation sets. The remaining empirical formulas are used exclusively for the test set, and this ensures that no molecules used during the training process have the same empirical formula as those used during testing. By separating the test set from the training and validation sets based on empirical formula, we have a stricter assessment of the model's performance when evaluating the model's predictions on the test set.

2. All the molecules that can potentially be used for the training and validation sets are gathered and organized by their empirical formulas. A specified variable indicates the maximum number of configurations to include for each empirical formula such that each empirical formula is represented as uniformly as possible. This is important because the ANI-1CCX dataset includes different numbers of configurations for different empirical formulas, and this number varies from one through several thousand. Standardizing the number of configurations for each empirical formula ensures that the

random sample is unbiased in favor of certain empirical formulas over others. Furthermore, the maximum number of configurations for each empirical formula is set such that the product between the number of empirical formulas and the maximum number of configurations for each formula is as close to the total number of training and validation molecules as possible.

3. The set of all possible molecules for the training and validation sets determined from step 2 is randomly shuffled. A random sampling without replacement is performed to obtain the set of training molecules followed by a second random sampling of the remaining molecules to obtain the set of validation molecules. The number of training molecules and the number of validation molecules is set such that the training molecules comprise 80% of the total and the validation molecules comprise 20%.

4. The test set is constructed systematically from the remaining empirical formulas such that every empirical formula has as equal as possible a number of configurations in the final set. This ensures that the test set gives a comprehensive representation of all the formulas included.

5. The test, train, and validation sets are saved to a specified directory and prepared for precomputation.

Once the base dataset is obtained, other datasets are generated as variations of this parent set. To generate larger datasets, the above workflow is repeated using the same empirical formulas as those found in the training set of the base dataset rather than randomly sampling from the original 471 formulas but with more configurations per formula and more molecules sampled in total. Both the training and validation sets are expanded when moving to datasets of larger size, but the test set is kept the same. To generate smaller datasets, a smaller training set is created by randomly sampling from the base dataset training set. For smaller datasets, both the validation and test sets remain unchanged.

For creating datasets with different partitioning schemes, the two we have focused on here are separating based on the number of heavy atoms and separating a larger dataset into two smaller datasets. When separating the data based on the number of heavy atoms, the above workflow is repeated except that the empirical formulas used for the training and validation sets are those containing fewer than or equal to some limit of heavy atoms and those used for the test set are those containing strictly greater than the limit of heavy atoms. This way, the training and validation sets are concerned solely with lighter molecules while the test set contains only the heavier molecules. When separating a larger dataset into two smaller datasets, the training set is split in half while the validation and test sets are copied over.

In terms of dealing with different physical targets, we are only concerned with varying the target used for the total molecular energy. In addition to assessing the performance of the model against CC level energies, we also wish to assess the performance of the model against DFT level energies. For this reason, a CC and DFT version of each dataset is generated for most of the experiments presented here, and the DFT version is usually copied over from the CC version with the only difference being the total energy value and the level of theory used.

S11 Outlier exclusion method

For removing outliers, the following process is applied on the predicted and target values for total molecular energy:

1. The absolute differences between the predicted total molecular energies and target total molecular energies, D_{ener}, are calculated.

2. The mean μ and standard deviation σ are calculated for the differences.

3. Because all values are positive, the number of standard deviations between the maximum value and the mean is calculated. If $\frac{max(D_{ener})-\mu}{\sigma} \geq 20$, the maximum value is removed from the set of differences.

4. Steps 2 and 3 are repeated until the set of differences is consistent and does not contain any values above 20 standard deviations from the mean.

Section 2.5 of the main manuscript compares performance of DFTBML and other models on the COMP6 benchmark suite. Table S16 lists the number of molecules that were outliers and the number of molecules that had SCF convergence errors in these tests. The format of Table S16 is similar to that of Table 6 of the main manuscript.

Table S16: Number of outliers and SCF convergence failures for various models, when applied to the COMP6 benchmark suite. The first number is the number of outliers and the second number, in parentheses, is the number of SCF convergence failures

**Parameterization**	**Ani MD**	**GDB**	**Drugbank**	**S66x8**	**Tripeptide**
Auorg	0(0)	0(5)	0(0)	0(0)	0(0)
MIO	0(0)	0(7)	0(0)	0(0)	0(0)
GFN1-xTB	0(256)	1(0)	0(192)	0(0)	0(0)
GFN2-xTB	0(256)	0(0)	0(48)	0(0)	0(0)
DFTBML CC	0(0)	0(3)	0(0)	0(0)	0(0)
Repulsive CC	0(0)	0(5)	0(0)	0(0)	0(0)
DFTBML DFT	0(0)	0(2)	0(0)	0(0)	0(0)
Repulsive DFT	0(0)	0(5)	0(0)	0(0)	0(0)
Transfer CC	0(0)	0(5)	0(0)	0(0)	0(0)
Transfer DFT	0(0)	0(3)	0(0)	0(0)	0(0)

S12 Hyperparameter investigation

Presented here is a detailed discussion of the hyperparameter sensitivity analyses. The following hyperparameters are of particular interest:

- **The weighting factors for the charges and dipoles:** The focus of DFTBML is to predict quantum chemical properties at a level of accuracy approaching that of *ab initio* CC theory. A great emphasis has been placed on predicting the total molecular energy, but also important is the performance of the model in predicting molecular dipoles and atomic charge. Different values of the weighting factors for these two physical targets are tested to determine optimal values. Because charge and dipole are coupled together, emphasis is placed on reproducing the observable quantities of total energy and molecular dipole.

- **The number of knots (control points) of the spline:** For splines, the knots or control points define the sequence of intervals where every interval is spanned by one polynomial function. Continuity conditions are enforced at these control points to ensure an overall continuous model.

- **The position of the inflection point:** The initial position of the inflection point for splines modeling overlap matrix elements. Inflection points were implemented as a way to account for the change in curvature of overlap integrals, a phenomenon most commonly observed when dealing with the overlap of two p orbitals.

- **The weighting factor w_{TD}:** One of the most important parameters for regularizing the functional form of these splines is the weighting factor for the penalty on the magnitude of the third derivative (see Section S6), as it is instrumental in ensuring the splines come out as smooth after training.

The hyperparameters chosen for each of these categories is as follows:

- **Energy, charge, and dipole weighting factors:** The following weighting factors (w_{prop} of Equation S25) are used: 6270 Ha^{-1} for total energy (Ha), 100 $(eÅ)^{-1}$ for dipoles (eÅ), and 1 e^{-1} for charges (e). Units are excluded in the subsequent discussion.

- **The number of knots for each spline:** The number of knots is set at 100.

- **The position of the inflection point:** The initial position of the inflection point is chosen to be 1/10 the total range of the spline.

- **The weighting factor for the third derivative penalty:** The factor is chosen to be $w_{TD} = 10$.

The following sections show, in detail, the effect that altering these hyperparameter values has on the performance of the model both quantitatively and qualitatively as it relates to model interpretability.

S12.1 The effect of the charge and dipole weighting factors

In addition to producing accurate predictions of the total molecular energy, DFTBML also aims to learn other quantities of interest. In total, predictions for three quantities are simultaneously optimized: total molecular energy, molecular dipole, and atomic charge. Internally, the dipole is calculated from the atomic charge by the following matrix multiplication:

$$\boldsymbol{\mu} = \mathbf{R}^T \mathbf{q} \tag{S37}$$

Where $\boldsymbol{\mu}$ is the dipole, $\mathbf{R}$ is a matrix of cartesian coordinates, and $\mathbf{q}$ is a vector of the atomic charges. In that sense, the atomic charge and dipole are coupled together. In early experiments with the DFTBML model, all three targets were trained at once with an independent weighting factor for each. This created problems as while the model was able to optimize all three targets, performance suffered as the three targets competed each other. While it is recognized that different weighting regimes can be used to tune the

accuracy for different targets, we have opted to focus on optimizing predictions of total molecular energy. Thus, a different approach is adopted where rather than introducing three hyperparameter weights with one per physical target, a greater emphasis is placed on the total energy and dipoles since these are observable quantities. Since charges and dipoles are linked, training the dipole prediction ability of DFTBML indirectly trains the ability to predict atomic charge.

To search for an optimal combination of weighting factors for the total energy and molecular dipole, the total energy was fixed with a weighting of 6270 and the charge was fixed with a weight of 1. The dipole weighting factor was then systematically varied over the value of 1, 10, 100, and 1000. The result of the experiments are shown in Figure S3.

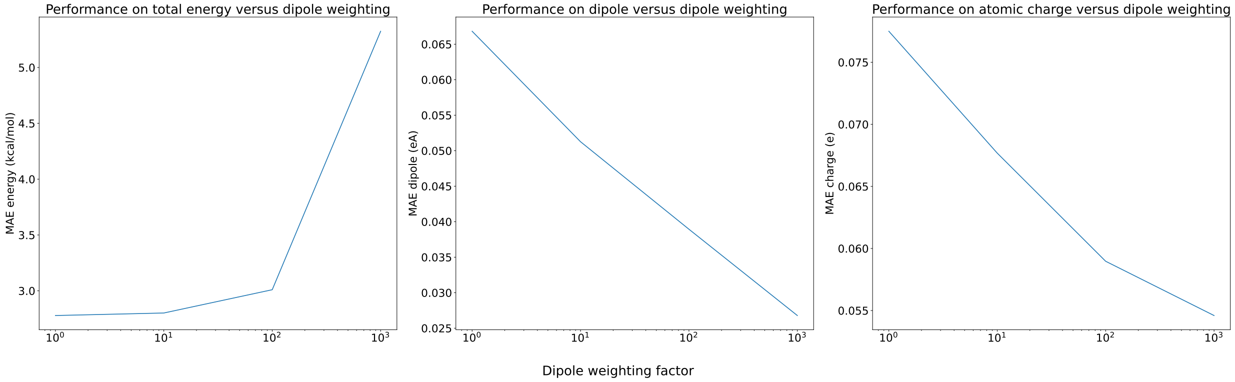

Figure S3: Performance on different physical targets as a function of the dipole weighting factor. The energy and charge weighting factors are fixed at 6270 and 1, respectively. The DFTBML CC 2500 dataset was used for the results presented here, and all the experiments were conducted for 1000 epochs with all other hyperparameters identical. These are the final numbers after any outliers have been excluded.

It is clear that increasing the dipole weighting factor leads to an improvement in the model's performance on dipoles and charges while a corresponding degradation in the model's performance on total energy is observed. Because the plots in Figure S3 are shown on a logarithmic scale, further analysis was done in the region from 10 to 100 and 100 to 1000 for the dipole weighting factor to confirm that the observed behavior was consistent. Figure S4 shows the results of testing the dipole weighting factor over the range of 200 to 900 with a step of 100 and Figure S5 shows the results of testing the dipole weighting factor over the

range of 20 to 90 with a step of 10.

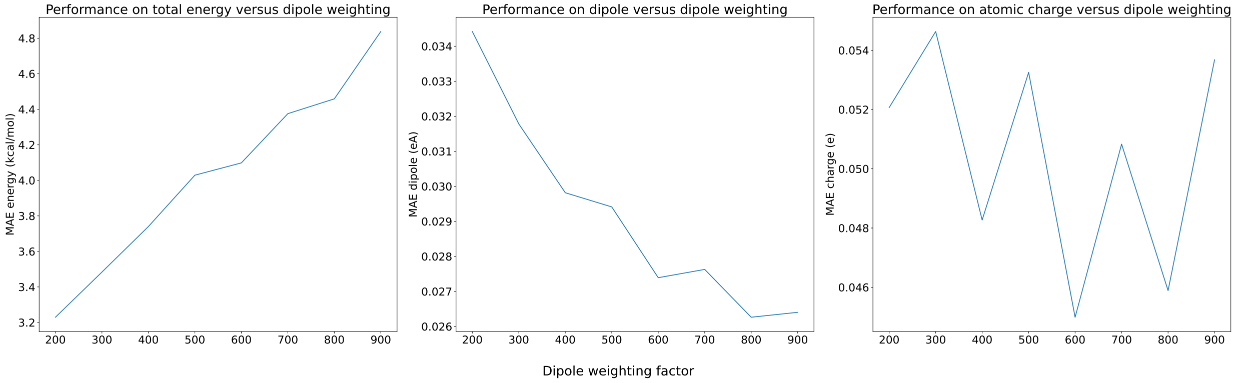

Figure S4: Performance on different physical targets as a function of the dipole weighting factor. The energy and charge weighting factors are fixed at 6270 and 1, respectively. The DFTBML CC 2500 dataset was used for the results presented here, and all the experiments were conducted for 1000 epochs with all other hyperparameters identical. These are the final numbers after any outliers have been excluded. The dipole weighting factor was scanned over the range of 200 to 900.

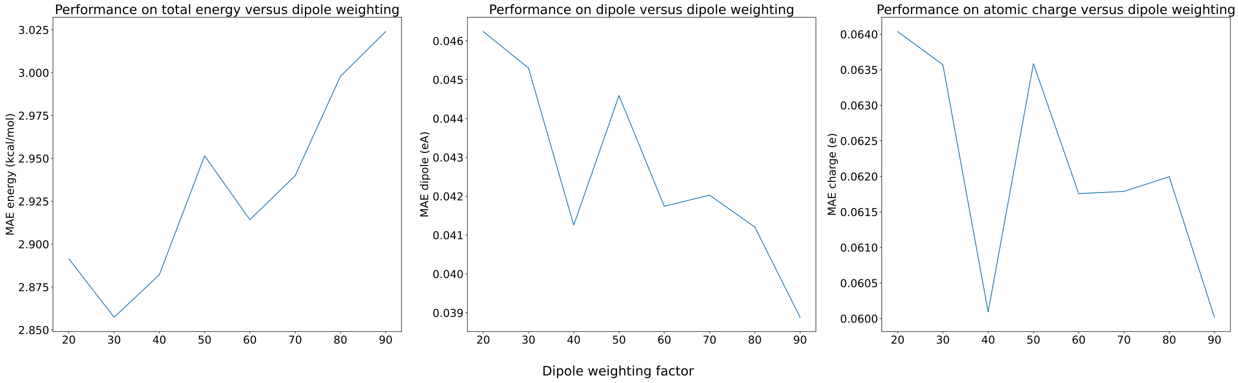

Figure S5: Performance on different physical targets as a function of the dipole weighting factor. The energy and charge weighting factors are fixed at 6270 and 1, respectively. The DFTBML CC 2500 dataset was used for the results presented here, and all the experiments were conducted for 1000 epochs with all other hyperparameters identical. These are the final numbers after any outliers have been excluded. The dipole weighting factor was scanned over the range of 20 to 90.

It is apparent that in the cases presented in Figures S4 and S5, the general trend shown in Figure S3 holds where reductions in the MAE for dipole and charges correspond with increases in the MAE for the total molecular energy. The erratic behavior of the charges in Figures S4 and S5 is not surprising because of how little weight is placed on charges during

the training process. No in-depth search was conducted over the interval spanning from 1 to 10 since increasing the dipole weighting factor by increments of 1 would not have a significant effect on the model's performance.

Based on this hyperparameter search, we use a weight of 6270 for total energy, 100 for dipole, and 1 for charges. A weighting factor of 100 is chosen for dipoles for two reasons. First, it was observed that increasing the weighting factor beyond 100 towards 1000 led to an increase in the number of outliers and the number of molecules which failed to converge on the SCF cycle of the DFTB+ program. While in each case the number of molecules which had to be removed because of non-convergence or exceeding the outlier threshold was less than 0.1%, the fact that the number of such occurrences increased with increasing dipole weight indicates the possibility of further instabilities from using parameters generated from this training approach. Second, a weight of 100 seems an optimal balance of performance for all three physical targets. Using a dipole weight of 100, the dipoles and charges can still be optimized to an extent without seeing a significant degradation in the model's performance on total energy. Furthermore, since going from a dipole weight of 1 or 10 to 100 does not result in a significant decrease in performance on total energy, it is worthwhile to use a relatively higher weight and gain some more performance on dipoles and charges rather than pursuing a marginal improvement in total energy. This investigation also motivated changing the number of epochs from 1000 to 2500 since 1000 epochs gave nearly full convergence of the targets but 2500 epochs was shown to give full convergence (see Section 2.3).

S12.2 The effect of the spline knot sequence

The number of knots chosen for the spline models define the number of intervals spanned by the polynomial basis functions such that for N knots, we have N - 1 intervals. The knots are initialized uniformly on the interval $[r_l, r_h]$ which defines the region over which the spline spans. Table S17 shows the results from using 25, 50, 75, 100, 125, and 150 knots for training to the CC total energy target, and Table S18 shows the results from using the

same numbers of knots but training to the DFT total energy target. All the experiments shown were conducted using 2500 epochs with a 2500 molecule dataset and the performance was evaluated on a near-transfer test set of 10000 molecules. No outliers were detected throughout.

Table S17: Performance of DFTBML models using different numbers of knots, when trained to the CC total energy target

Parameterization	Nonconverged	MAE energy (kcal/mol)	MAE dipole (eÅ)	MAE charge (e)
DFTBML CC 2500, 150 knots	0	3.12	0.039	0.059
DFTBML CC 2500, 125 knots	0	3.00	0.037	0.057
DFTBML CC 2500, 100 knots	0	3.00	0.039	0.059
DFTBML CC 2500, 75 knots	1	2.92	0.036	0.058
DFTBML CC 2500, 50 knots	1	2.86	0.036	0.059
DFTBML CC 2500, 25 knots	0	2.83	0.036	0.056

Table S18: Performance of DFTBML models using different numbers of knots, when trained to the DFT total energy target

Parameterization	Nonconverged	MAE energy (kcal/mol)	MAE dipole (eÅ)	MAE charge (e)
DFTBML DFT 2500, 150 knots	0	3.11	0.039	0.057
DFTBML DFT 2500, 125 knots	0	3.08	0.040	0.055
DFTBML DFT 2500, 100 knots	1	3.03	0.038	0.056
DFTBML DFT 2500, 75 knots	0	2.93	0.034	0.045
DFTBML DFT 2500, 50 knots	1	2.98	0.037	0.052
DFTBML DFT 2500, 25 knots	0	2.92	0.037	0.058

Numerically, the performance across the different numbers of knots is similar in terms of both total energy and dipole, although some deviations are observed for predicting the atomic charge. However, this is likely because the hyperparameters were set such that the optimization focus was on the total molecular energy. Figure S6 shows the overlay of a series of splines used to model two different overlap matrix elements.

An interesting observation is that there is a clear difference in behavior between splines having 75 and fewer knots and those splines with 100 or more knots, whereby the splines with 75 or fewer knots have a tendency to resort to longer range interactions while also precluding the upward inflection at short range. In contrast to this, splines with 100 or more knots tend to keep interactions in the short range and are able to include the upward inflection in the functional forms. Furthermore, splines with 100+ knots all tend to give the

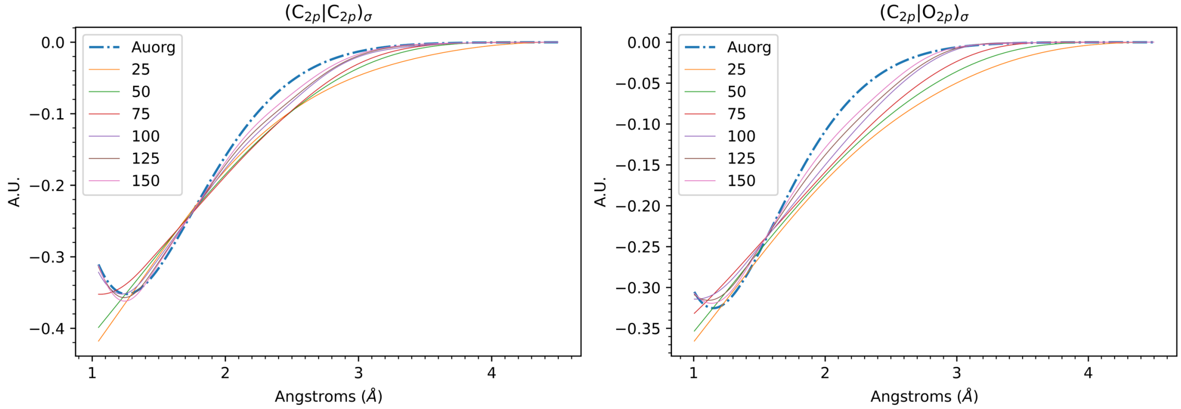

Figure S6: Overlay of spline models with different numbers of knots. The carbon-oxygen 2p overlap interaction is shown on the right and the carbon-carbon 2p overlap interaction is shown on the left. Both interactions are σ in orientation and both plots are generated from the DFTBML CC results. Auorg curves are included for reference.

same final form with close agreement, and their physical form more closely resembles that of the Auorg potentials, especially at short range. Based on the results here, it is clear that 100 knots is the optimal number since it is the minimal number of knots required to produce physically intuitive short range functional forms that incorporate an upward inflection and which incorporate features of the Auorg parameterization.

S12.3 The effect of the inflection point position

For DFTBML, an inflection point follows the mathematical definition in that the curvature (i.e., sign of the second derivative) changes across the point. The implementation of the inflection point penalty is described in Section S6.

Because the inflection point variable is tied to the convex penalty and because the convex penalty converges quickly to 0 due to the nature of the inequality constraint, the inflection point shows little motility during training, typically moving less than 0.1 Å from its starting point. Because of this, the initial value of the inflection point is considered a hyperparameter

and is specified as some fraction of the range from r_l to r_h as follows:

$$r_{inflect} = r_l + \left(\frac{r_h - r_l}{x}\right) \tag{S38}$$

Where the denominator x is varied, i.e. if the target is 1/10 the range, then x is set to 10. To determine an optimal value of this hyperparameter, a series of near-transfer experiments were conducted including no inflection point, inflection point initialized at 1/15 the range, inflection point initialized at 1/10 the range, and inflection point initialized at 1/5 the range. The results of these experiments are shown in Table S19, where each experiment was run for 2500 epochs and both CC and DFT energy targets were analyzed. Each experiment used a test set of 10000 molecules. Figure S7 shows the overlaid results of these different inflection point runs for the $(C_{2p}|C_{2p})_\sigma$ and $(C_{2p}|N_{2p})_\sigma$ integrals where the upward curvature at short distances is evident.

Table S19: Performance of the model using different initial values for the inflection point

Parameterization	Nonconverged	MAE energy (kcal/mol)	MAE dipole (eÅ)	MAE charge (e)
DFTBML CC 2500, no inflection	0	3.13	0.039	0.059
DFTBML CC 2500, x = 5	0	3.03	0.035	0.058
DFTBML CC 2500, x = 10	0	3.00	0.039	0.059
DFTBML CC 2500, x = 15	1	3.02	0.038	0.061
DFTBML DFT 2500, no inflection	0	3.21	0.040	0.054
DFTBML DFT 2500, x = 5	0	3.12	0.038	0.057
DFTBML DFT 2500, x = 10	1	3.03	0.038	0.056
DFTBML DFT 2500, x = 15	1	3.03	0.036	0.055

As observed in Table S19, variations in the starting position of the inflection point has no major impact on the model's performance so long as it is initialized at relatively short range. This makes sense since in the short range region around 1.5 Å, the curvature of the model is nearly zero, and so moving the inflection point along a region without changing curvature does not have an effect. In Figure S7, we can see that for the cases of x = 5, 10, or 15 for the inflection point initial value, the functional forms are fairly similar. However, the case of no inflection point differs dramatically in that no upward curvature is allowed at shorter range. Since the positioning of the inflection point does not have a significant impact on the

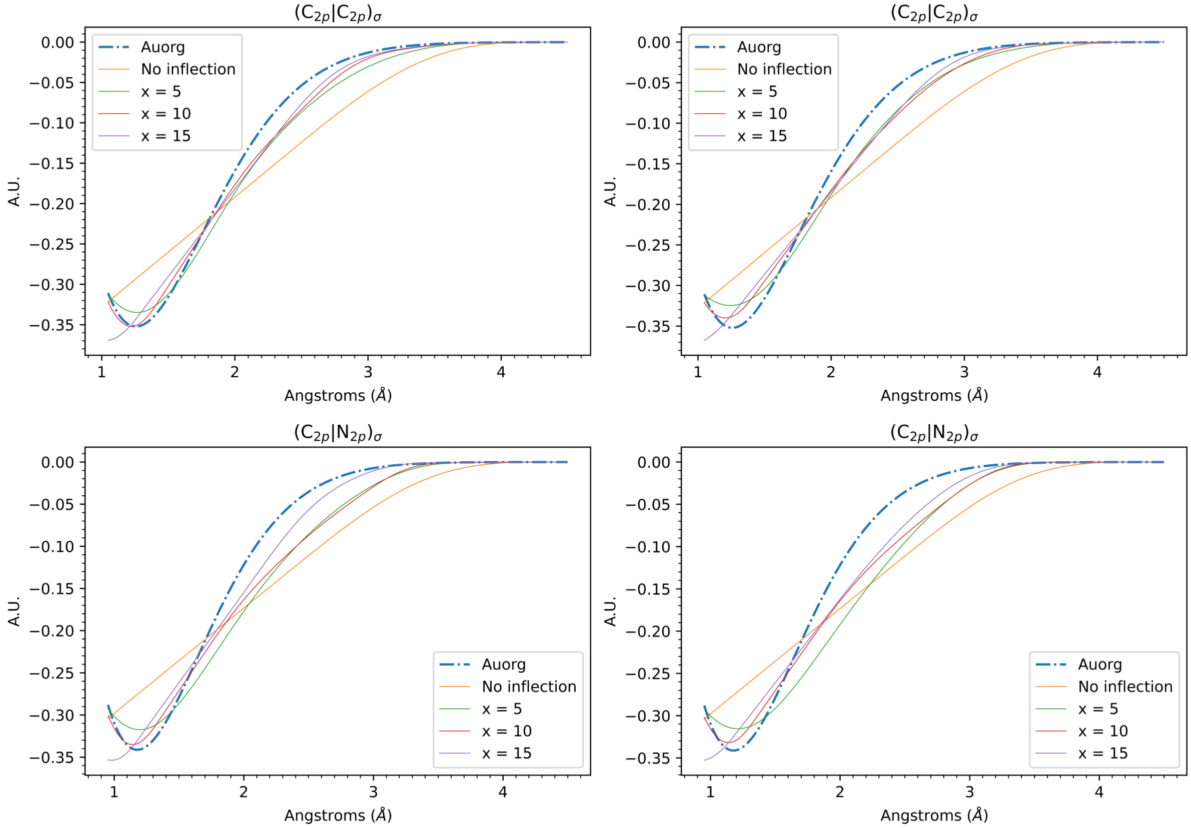

Figure S7: Spline models for the $(C_{2p}|C_{2p})_\sigma$ and $(C_{2p}|N_{2p})_\sigma$ integrals, trained using different initial values for the inflection point. The left column is from training to the CC total energy target and the right column is from training to the DFT total energy target.

model's performance, an inflection point initialization value of 1/10 is set as the standard for all experiments.

S12.4 The effect of the third derivative penalty weight

For the third derivative penalty, a weighting factor is applied to control how aggressively the magnitude of the third derivative is penalized. The mathematical form of the penalty is given in Equation S28. The effect of the weighting factor w_{TD} was investigated by systematically varying the value of w_{TD} by multiples of 10. In total, six values were tested, with w_{TD} = 0, 0.1, 1, 10, 100, and 1000. Tables S20 and S21 below show the results for different experiments conducted with these different hyperparameter values and Figure S8

shows some resulting spline models. All the experiments conducted used 2500 epochs, 6270 for the energy weighting factor, 100 for the dipole weighting factor, 1 for the charge weighting factor, 100 knots, and an inflection point initialization value of 1/10 the total range. The test set consisted of 10000 molecules.

Table S20: Performance of the model trained to CC total energy using different weight values for the third derivative penalty

Parameterization	Outliers	Nonconverged	MAE energy (kcal/mol)	MAE dipole (eÅ)	MAE charge (e)
DFTBML CC 2500 $w_{TD} = 0$	0	0	3.17	0.041	0.060
DFTBML CC 2500 $w_{TD} = 0.1$	0	0	3.17	0.039	0.057
DFTBML CC 2500 $w_{TD} = 1$	0	0	3.05	0.040	0.060
DFTBML CC 2500 $w_{TD} = 10$	0	0	3.00	0.039	0.059
DFTBML CC 2500 $w_{TD} = 100$	0	1	3.69	0.037	0.058
DFTBML CC 2500 $w_{TD} = 1000$	0	1	5.60	0.043	0.061

Table S21: Performance of the model trained to DFT total energy using different weight values for the third derivative penalty

Parameterization	Outliers	Nonconverged	MAE energy (kcal/mol)	MAE dipole (eÅ)	MAE charge (e)
DFTBML DFT 2500 $w_{TD} = 0$	1	0	3.17	0.039	0.056
DFTBML DFT 2500 $w_{TD} = 0.1$	1	0	3.04	0.036	0.044
DFTBML DFT 2500 $w_{TD} = 1$	0	0	3.13	0.041	0.057
DFTBML DFT 2500 $w_{TD} = 10$	0	1	3.03	0.038	0.056
DFTBML DFT 2500 $w_{TD} = 100$	0	0	3.78	0.039	0.058
DFTBML DFT 2500 $w_{TD} = 1000$	0	1	5.29	0.042	0.060

It is evident from Tables S20 and S21 that variations of the w_{TD} have no significant effect at lower values, but does significantly degrade model performance at the higher values of 100 and 1000. However, using values lower than 10 for the weighting factor does lead to the emergence of piecewise-linear behavior in the model which is undesirable, as seen in Figure S8. Taking this into account, $w_{TD} = 10$ is the optimal choice out of those tested, and it is the standard value used for experiments.

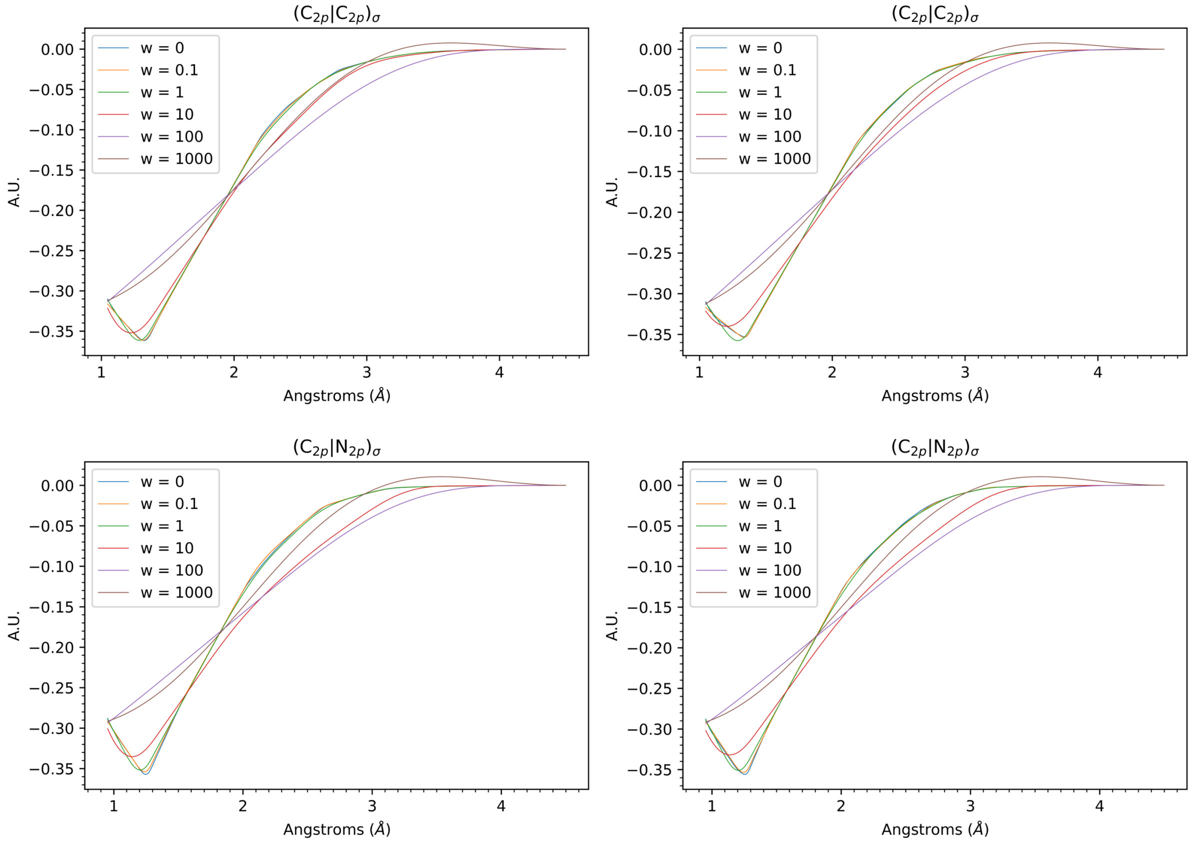

Figure S8: Spline models for the $(C_{2p}|C_{2p})_\sigma$ and $(C_{2p}|N_{2p})_\sigma$ integrals, trained using different weight values for the third derivative penalty. The left column is from training to the CC total energy target and the right column is from training to the DFT total energy target.

S13 Tables and figures for Section 2

S13.1 A note on learning curves

In this section is presented the learning curves for all of the experiments performed for this study. The per-batch loss, as shown in Equation S25 in Section S6, is the quantity on which gradient descent steps are performed after every batch. However, the learning curves here report the average epoch loss, i.e. the loss averaged over the number of batches for the training and validation data for a given epoch, respectively, separated by target. Thus for a given target (i.e. total energy, dipoles, or charges), the values reported in the loss curves is as follows:

$$L_{prop_k} = \frac{1}{N_{batch}} \sum_{j}^{N_{batch}} \sqrt{\frac{1}{N_{prop_j}} \sum_{i}^{N_{prop_j}} |Pred_{i,j} - Target_{i,j}|^2}, \forall prop \tag{S39}$$

Where L_{prop_k} is the loss for the property $prop$ on epoch k. N_{batch} is the number of batches in a given epoch, and the remaining terms have the same meaning as in Equation S25. Due to the nature of its construction, this loss term gives back the appropriate units for each physical target, and the weighting factors w_{prop} are divided out prior to reporting in the loss curves. The total energy loss is reported per heavy atom since internally, DFTBML trains on total energy per heavy atom as a way to account for the effect of molecule size.

S13.2 Repulsive potential performance

It is worthwhile to see how the repulsive potential performs on its own and to quantify the benefit of training the electronic portion of the model. For training the repulsive potential, the same datasets used for the experiments in Section 2.3 are repurposed, and the target that the repulsive model is being trained to is the difference between the true total molecular energy and the predicted electronic energy of the molecule obtained from a DFTB calculation using the Auorg parameters (see Section S4).

The naming conventions are established in Section 2.1 of the main paper. Full results can be seen in Tables S22, S23, and S24. All the experiments conducted used cubic splines with 50 knots and a vanishing boundary condition (zero, first, and second derivative all go to 0 at r_h). The results are also presented on a per heavy atom and per atom basis in Tables S25, S26, and S27.

Table S22: Performance of the repulsive potential trained to the CC total energy target

Parameterization	Outliers	Nonconverged	MAE energy (kcal/mol)	MAE dipole (eÅ)	MAE charge (e)
Auorg	0	0	10.55	0.079	0.085
MIO	0	0	10.69	0.079	0.085
GFN1-xTB	0	0	10.66	0.136	0.103
GFN2-xTB	0	0	13.03	0.153	0.089
Repulsive CC 20000	3	0	5.41	0.079	0.085
Repulsive CC 10000	4	0	5.41	0.079	0.085
Repulsive CC 5000	0	0	5.41	0.079	0.085
Repulsive CC 2500	0	0	5.42	0.079	0.085
Repulsive CC 1000	2	0	5.42	0.079	0.085
Repulsive CC 300	3	0	5.96	0.079	0.085

Table S23: Performance of the repulsive potential trained to the DFT total energy target

Parameterization	Outliers	Nonconverged	MAE energy (kcal/mol)	MAE dipole (eÅ)	MAE charge (e)
Auorg	0	0	11.95	0.079	0.085
MIO	0	0	11.69	0.079	0.085
GFN1-xTB	0	0	9.83	0.136	0.103
GFN2-xTB	0	0	11.82	0.153	0.089
Repulsive DFT 20000	0	0	5.71	0.079	0.085
Repulsive DFT 10000	0	0	5.75	0.079	0.085
Repulsive DFT 5000	0	0	5.74	0.079	0.085
Repulsive DFT 2500	0	0	5.74	0.079	0.085
Repulsive DFT 1000	3	0	5.74	0.079	0.085
Repulsive DFT 300	3	0	6.51	0.079	0.085

Table S24: Performance of the repulsive potential in far-transfer

Parameterization	Outliers	Nonconverged	MAE energy (kcal/mol)	MAE dipole (eÅ)	MAE charge (e)
Auorg CC	0	0	11.81	0.089	0.088
MIO CC	0	0	11.86	0.089	0.088
Auorg DFT	0	0	13.25	0.089	0.088
MIO DFT	0	0	13.05	0.089	0.088
GFN1-xTB CC	0	0	10.85	0.147	0.104
GFN2-xTB CC	0	0	13.51	0.165	0.091
GFN1-xTB DFT	0	0	10.14	0.147	0.104
GFN2-xTB DFT	0	0	12.26	0.165	0.091
Repulsive Transfer CC 2500	0	0	7.17	0.089	0.088
Repulsive Transfer DFT 2500	0	0	7.39	0.089	0.088

Table S25: Performance per atom and per heavy atom of the repulsive potential trained to the CC total energy target

**Parameterization**	**MAE energy (kcal mol$^{-1}N_{heavy}^{-1}$)**	**MAE energy (kcal mol$^{-1}N_{atom}^{-1}$)**
Auorg	1.71	0.97
MIO	1.74	0.98
GFN1-xTB	1.83	1.05
GFN2-xTB	2.23	1.33
Repulsive CC 20000	0.95	0.54
Repulsive CC 10000	0.94	0.54
Repulsive CC 5000	0.94	0.54
Repulsive CC 2500	0.94	0.54
Repulsive CC 1000	0.96	0.55
Repulsive CC 300	1.02	0.58

Table S26: Performance per atom and per heavy atom of the repulsive potential trained to the DFT total energy target

**Parameterization**	**MAE energy (kcal mol$^{-1}N_{heavy}^{-1}$)**	**MAE energy (kcal mol$^{-1}N_{atom}^{-1}$)**
Auorg	1.92	1.10
MIO	1.88	1.07
GFN1-xTB	1.71	0.97
GFN2-xTB	2.05	1.21
Repulsive DFT 20000	1.01	0.57
Repulsive DFT 10000	1.02	0.58
Repulsive DFT 5000	1.00	0.57
Repulsive DFT 2500	1.00	0.57
Repulsive DFT 1000	1.02	0.58
Repulsive DFT 300	1.12	0.64

Table S27: Performance per atom and per heavy atom of the repulsive potential in far-transfer

**Parameterization**	**MAE energy (kcal mol$^{-1}N_{heavy}^{-1}$)**	**MAE energy (kcal mol$^{-1}N_{atom}^{-1}$)**
Auorg CC	1.64	0.97
MIO CC	1.65	0.97
Auorg DFT	1.85	1.10
MIO DFT	1.82	1.07
GFN1-xTB CC	1.52	0.91
GFN2-xTB CC	1.88	1.16
GFN1-xTB DFT	1.41	0.83
GFN2-xTB DFT	1.71	1.03
Repulsive Transfer CC 2500	0.99	0.60
Repulsive Transfer DFT 2500	1.02	0.61

Unlike when training the electronic model, the only target of interest here is the performance on total molecular energy since training the repulsive potential only introduces an energy correction term. As seen in Tables S22 and S23, the MAE on dipoles and atomic charges remains consistent despite the change in the size of the dataset. An additional observation is that the improvement in performance for total energy saturates quickly and there are no significant improvements obtained by consistently increasing the amount of training data beyond 1000 molecules. In training to either the CC or DFT total energy target, the performance from using 2500 through 20000 training molecules remains remarkably steady, leveling out around 5.4 kcal/mol in the CC case and 5.7 kcal/mol in the DFT case. A degradation of performance associated with a lack of training data is only observed when dropping to 300 training molecules in both cases.

In terms of the repulsive potential's performance in the far-transfer experiments, the repulsive potential alone performs worse than the full DFTBML model (see Table 3 of the main paper), but it still performs better than the xTB methods and standard DFTB parameterizations. Collectively, the results presented show that training only the repulsive potential does provide an improvement to the performance of base DFTB, but it does not perform as well as the full DFTBML model. Furthermore, the repulsive potential is not as sensitive to the quantity of training data as the full model. This may be partly related to the use of a quadratic programming approach to find the global minimum, as opposed to the gradient descent approach used to train the full DFTBML model.

The functional forms of the models used for the repulsive potential are simpler than the forms for the models used for the Hamiltonian or overlap integrals since the repulsive potential is a classical interaction intended to include interactions between the core electrons that are excluded from the DFTB Hamiltonian. As such, the potentials are constrained to have a positive second derivative at all points, giving a smooth and exponentially decaying form. A few examples of the repulsive potential are shown in Figure S9. In total there are 10 such potentials needed to specify all pairwise interactions between only C, H, N, and O

atoms.

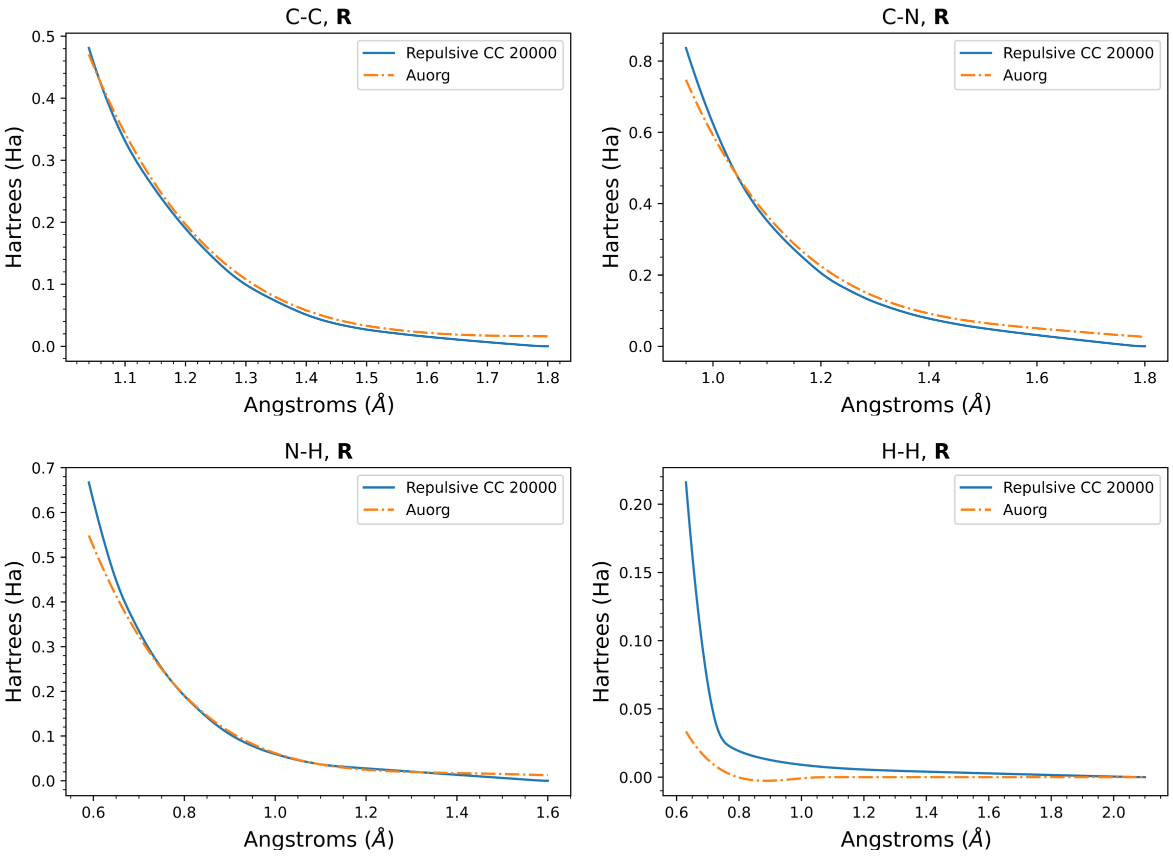

Figure S9: Four different repulsive potentials from the Repulsive CC 20000 experiment. The Auorg repulsive potentials are overlaid for reference.

S13.3 Unregularized model performance

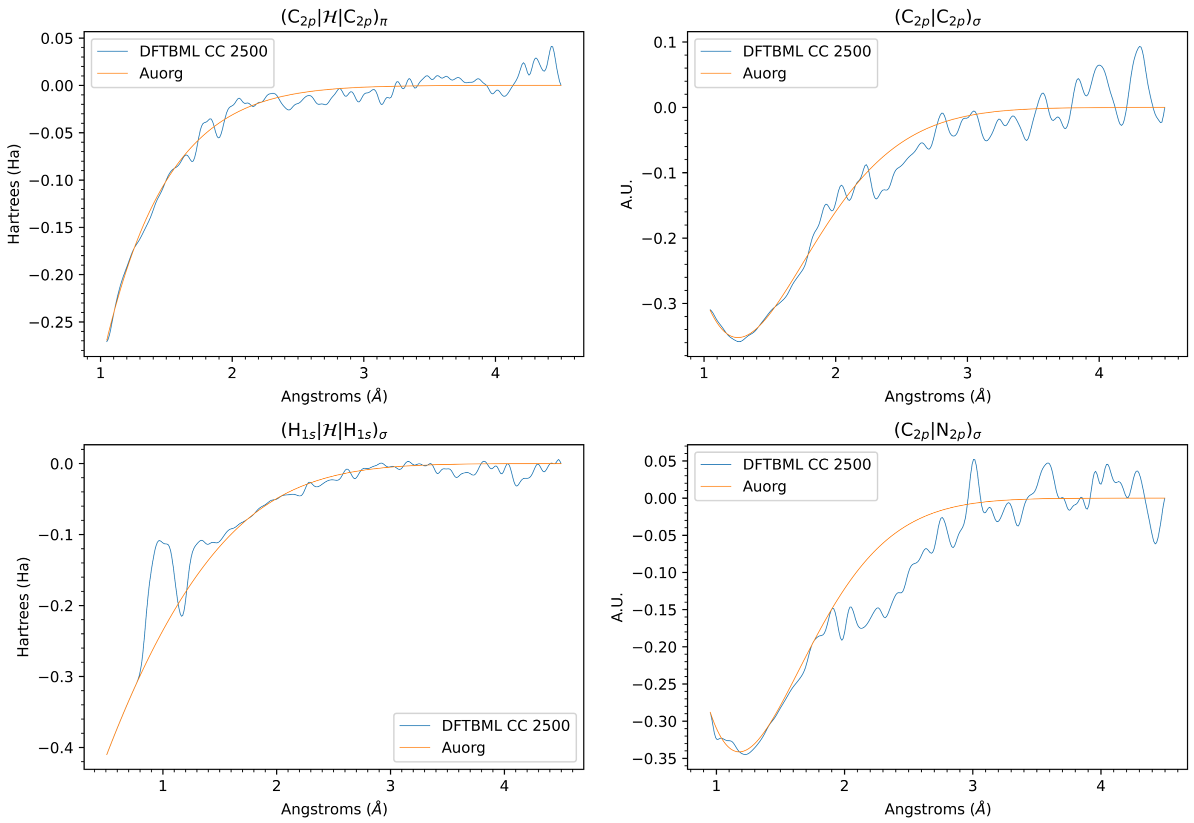

Figure S10: Examples of unregularized splines obtained from training using the DFTBML CC 2500 dataset. The Auorg potentials are included for reference.

S13.4 Model performance when training on different dataset sizes

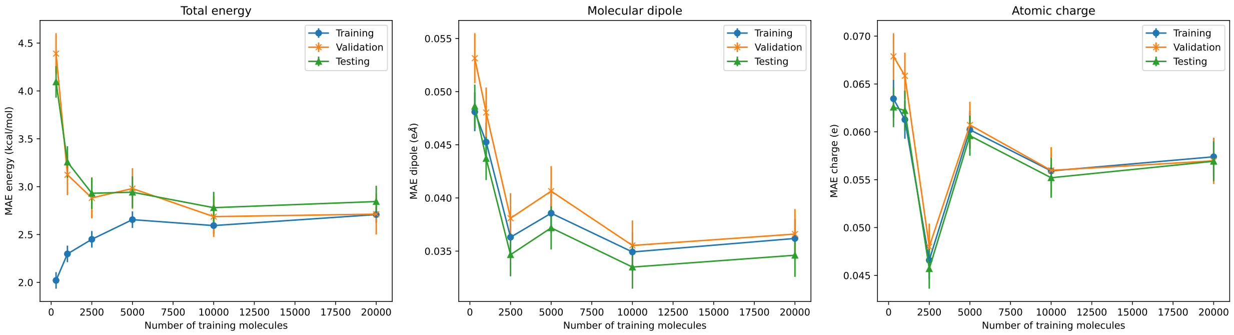

Figure S11: Final training, validation, and test loss for each of the physical targets as a function of the size of the dataset used for training. Results were obtained using the datasets containing the DFT total energy targets. Error bars are shown as $\pm\frac{\sigma}{3}$ where σ is the standard deviation of the errors calculated separately for the training, validation, and testing values.

Table S28: Performance of different parameterizations trained against CC energy target

Parameterization	Outliers	Nonconverged	MAE energy (kcal/mol)	MAE dipole (eÅ)	MAE charge (e)
Auorg	0	0	10.55	0.079	0.085
MIO	0	0	10.69	0.079	0.085
GFN1-xTB	0	0	10.66	0.136	0.103
GFN2-xTB	0	0	13.03	0.153	0.089
DFTBML CC 20000	0	0	2.67	0.033	0.053
DFTBML CC 10000	0	0	2.68	0.031	0.044
DFTBML CC 5000	0	1	2.84	0.035	0.055
DFTBML CC 2500	0	1	2.95	0.036	0.054
DFTBML CC 1000	2	0	3.25	0.041	0.058
DFTBML CC 300	4	0	4.14	0.052	0.066

Table S29: Performance of different parameterizations trained against DFT energy target

Parameterization	Outliers	Nonconverged	MAE energy (kcal/mol)	MAE dipole (eÅ)	MAE charge (e)
Auorg	0	0	11.95	0.079	0.085
MIO	0	0	11.69	0.079	0.085
GFN1-xTB	0	0	9.83	0.136	0.103
GFN2-xTB	0	0	11.82	0.153	0.089
DFTBML DFT 20000	0	0	2.84	0.035	0.057
DFTBML DFT 10000	0	0	2.78	0.033	0.055
DFTBML DFT 5000	0	0	2.94	0.037	0.060
DFTBML DFT 2500	0	0	2.93	0.035	0.046
DFTBML DFT 1000	3	0	3.26	0.044	0.062
DFTBML DFT 300	5	0	4.09	0.049	0.063

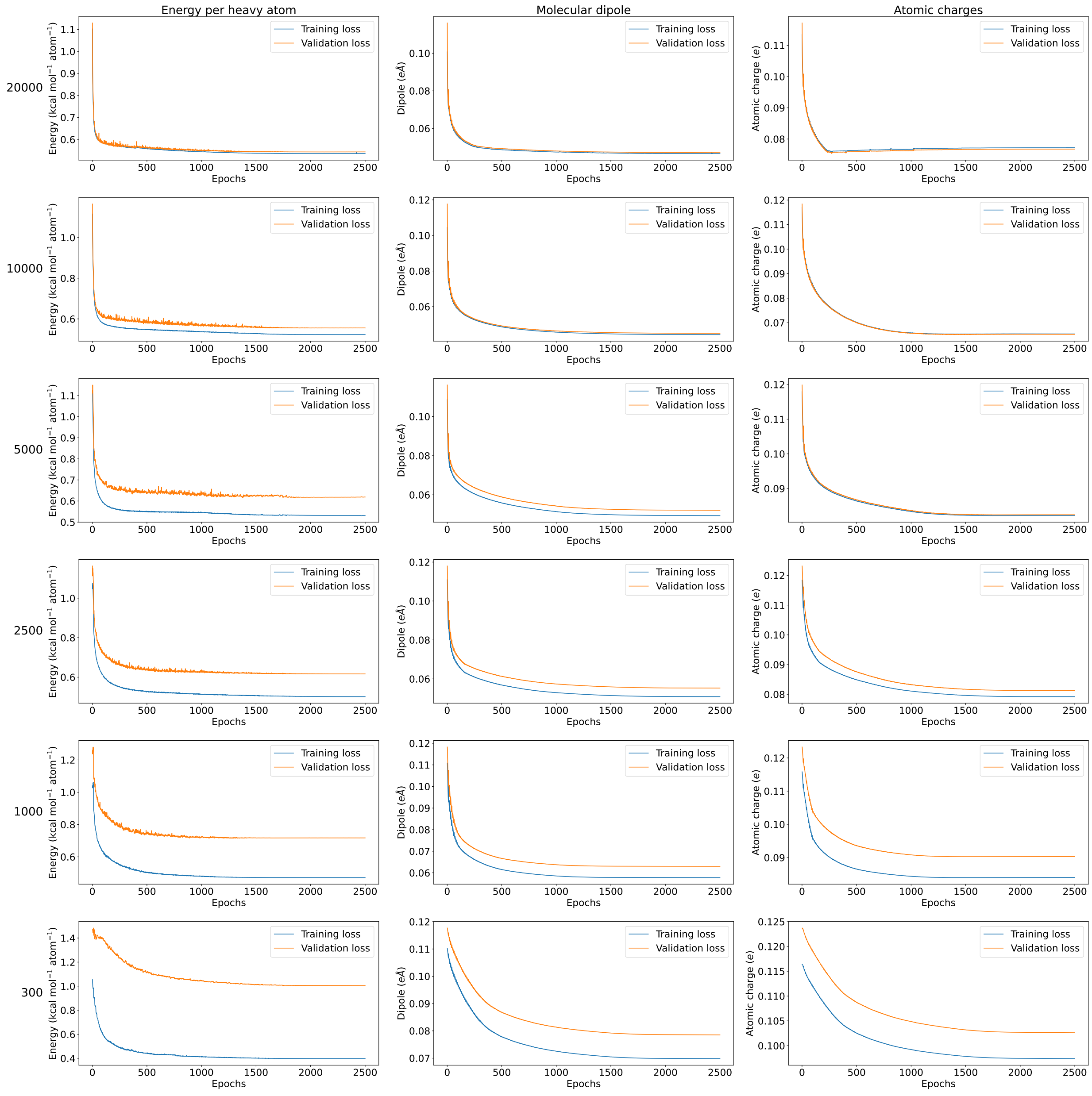

Figure S12: Learning curves for total energy per heavy atom, molecular dipole, and atomic charge when training to the CC energy target for total energy. The label for each row indicates the number of training molecules used for each experiment.

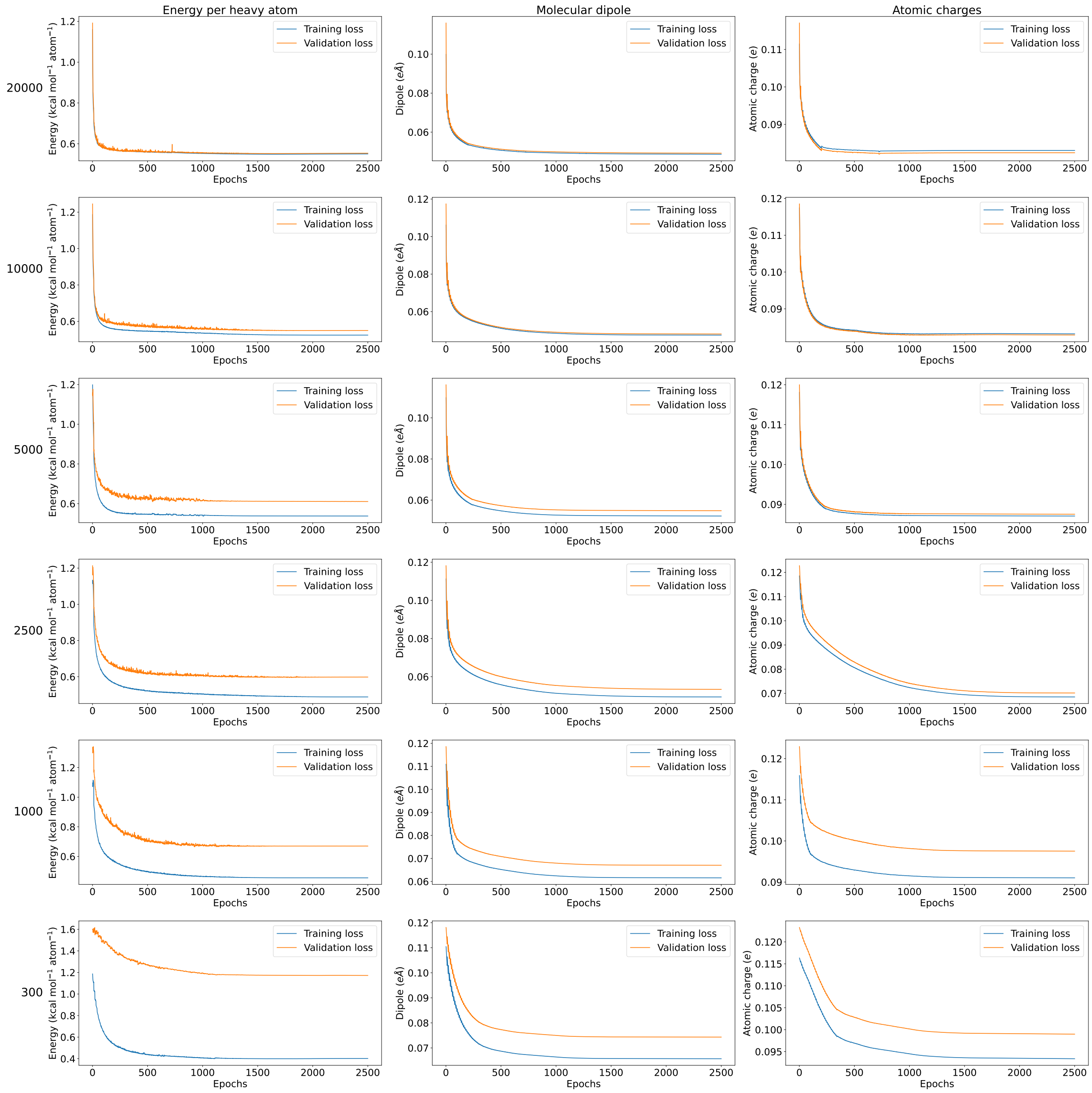

Figure S13: Learning curves for total energy per heavy atom, molecular dipole, and atomic charge when training to the DFT energy target for total energy. The label for each row indicates the number of training molecules used for each experiment.

Table S30: MAE in energy per atom and per heavy atom when trained against CC total energy targets

Parameterization	MAE energy (kcal $mol^{-1}N_{heavy}^{-1}$)	MAE energy (kcal $mol^{-1}N_{atom}^{-1}$)
Auorg	1.71	0.97
MIO	1.74	0.98
GFN1-xTB	1.83	1.05
GFN2-xTB	2.23	1.33
DFTBML CC 20000	0.44	0.25
DFTBML CC 10000	0.45	0.26
DFTBML CC 5000	0.47	0.26
DFTBML CC 2500	0.49	0.28
DFTBML CC 1000	0.56	0.32
DFTBML CC 300	0.71	0.40

Table S31: MAE in energy per atom and per heavy atom when trained against DFT total energy targets

Parameterization	MAE energy (kcal $mol^{-1}N_{heavy}^{-1}$)	MAE energy (kcal $mol^{-1}N_{atom}^{-1}$)
Auorg	1.92	1.10
MIO	1.88	1.07
GFN1-xTB	1.71	0.97
GFN2-xTB	2.05	1.21
DFTBML DFT 20000	0.48	0.27
DFTBML DFT 10000	0.46	0.26
DFTBML DFT 5000	0.49	0.28
DFTBML DFT 2500	0.49	0.28
DFTBML DFT 1000	0.57	0.33
DFTBML DFT 300	0.71	0.40

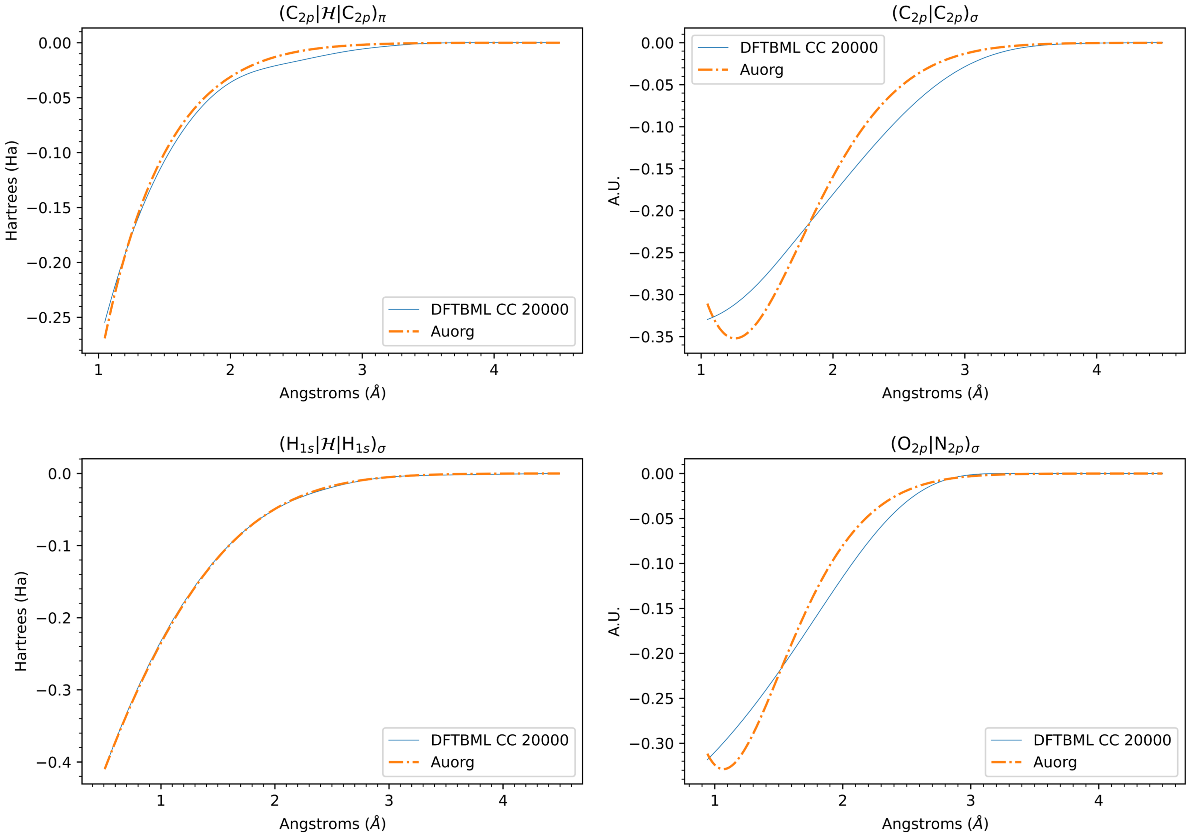

Figure S14: Representative splines generated from DFTBML CC 20000 for a few of the matrix elements involved in computing properties for organic molecules. The two plots on the left hand column involve Hamiltonian matrix elements and the two plots on the right hand column detail overlap matrix elements. σ and π are used to indicate the orientation of the interaction. Hamiltonian matrix elements are given in units of Hartrees and the overlap matrix elements have arbitrary units (A.U.). The potential functions from the Auorg reference set are overlaid for comparison.

S13.5 Model transferability and reproducibility

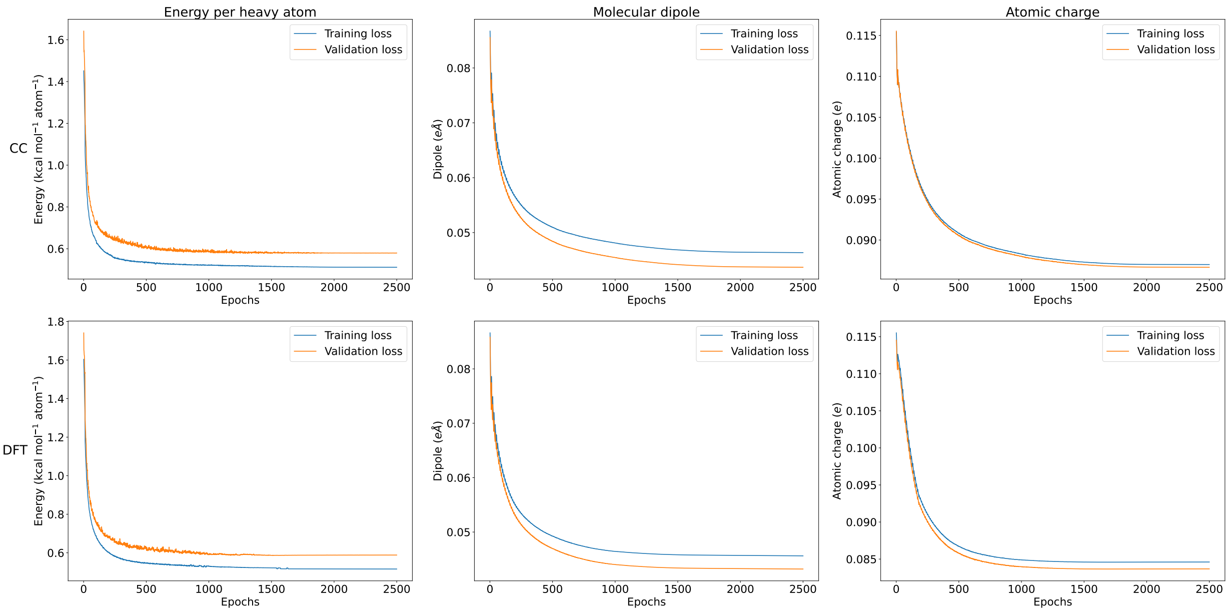

Figure S15: Learning curves for total energy per heavy atom, molecular dipole, and atomic charge for far-transfer training. The top row is for training to the CC total energy target and the bottom row is for training to the DFT total energy target.

Table S32: MAE in energy per atom and per heavy atom in far-transfer

**Parameterization**	**MAE energy (kcal mol^{-1}N_{heavy}^{-1})**	**MAE energy (kcal mol^{-1}N_{atom}^{-1})**
Auorg CC	1.64	0.97
MIO CC	1.65	0.97
Auorg DFT	1.85	1.10
MIO DFT	1.82	1.07
GFN1-xTB CC	1.52	0.91
GFN2-xTB CC	1.88	1.16
GFN1-xTB DFT	1.41	0.83
GFN2-xTB DFT	1.71	1.03
Transfer CC 2500	0.66	0.41
Transfer DFT 2500	0.66	0.41

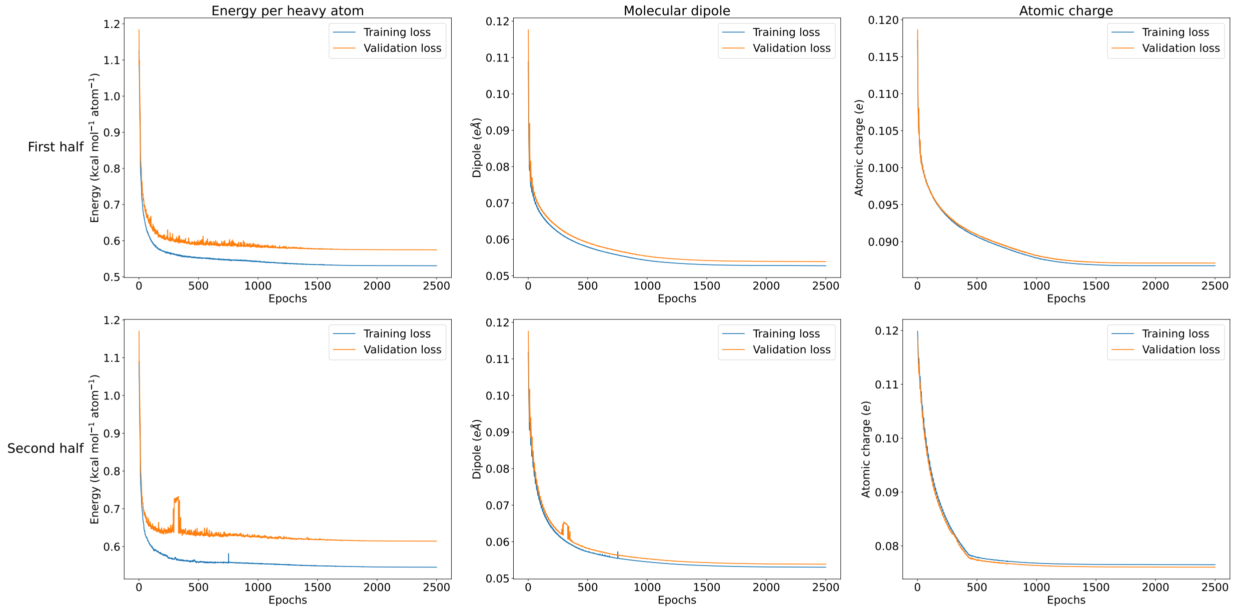

Figure S16: Learning curves for total energy per heavy atom, molecular dipole, and atomic charge from assessing model reproducibility from two disjoint training sets.

Table S33: MAE in energy per atom and per heavy atom trained on two disjoint training sets

**Parameterization**	**MAE energy (kcal mol$^{-1}N_{heavy}^{-1}$)**	**MAE energy (kcal mol$^{-1}N_{atom}^{-1}$)**
Auorg	1.71	0.97
MIO	1.74	0.98
GFN1-xTB	1.83	1.05
GFN2-xTB	2.23	1.33
DFTBML CC 5000 First Half	0.46	0.26
DFTBML CC 5000 Second Half	0.49	0.28

S13.6 DFTBML performance on COMP6 benchmark

Table S34: MAE total energy in kcal/mol for DFTBML and various models on COMP6 benchmark suite

Test set	Auorg	MIO	GFN1-xTB	GFN2-xTB	DFTBML CC	Repulsive CC	DFTBML DFT	Repulsive DFT	Transfer CC	Transfer DFT
Ani MD	6.23	5.78	4.80	7.94	5.13	6.56	4.48	5.69	5.30	**4.17**
Drugbank	13.39	12.66	11.40	10.97	8.14	10.78	**6.51**	9.57	8.74	6.71
GDB 7	9.33	9.02	8.40	7.64	3.84	6.26	**3.17**	5.68	4.32	3.43
GDB 8	10.20	9.89	8.99	8.09	4.06	6.43	**3.29**	5.92	4.56	3.53
GDB 9	10.99	10.66	8.96	8.62	4.38	7.37	**3.44**	6.59	4.99	3.72
GDB 10	10.70	10.45	9.79	9.18	4.58	7.17	**3.67**	6.47	5.29	3.96
GDB 11	12.69	12.51	10.32	10.66	5.83	8.83	**4.11**	7.92	6.66	4.49
GDB 12	13.70	13.53	10.59	11.03	6.18	9.65	**4.21**	8.44	6.71	4.46
GDB 13	14.10	13.91	11.37	11.44	6.52	10.09	**4.38**	8.85	7.25	4.72
S66x8	4.28	3.65	4.68	4.65	2.64	4.52	**2.64**	4.54	3.90	2.94
Tripeptide	9.11	8.96	6.25	7.36	5.04	7.74	**4.02**	6.23	6.11	4.83

Table S35: MAE dipole in eÅ for DFTBML and various models on COMP6 benchmark suite

Test set	Auorg	MIO	GFN1-xTB	GFN2-xTB	DFTBML CC	Repulsive CC	DFTBML DFT	Repulsive DFT	Transfer CC	Transfer DFT
Ani MD	0.167	0.168	0.170	0.205	**0.104**	0.167	0.106	0.167	0.119	0.115
Drugbank	0.111	0.111	0.207	0.242	**0.053**	0.111	0.055	0.111	0.066	0.064
GDB 7	0.088	0.088	0.138	0.157	**0.031**	0.088	0.033	0.088	0.045	0.043
GDB 8	0.094	0.094	0.144	0.163	**0.034**	0.094	0.036	0.094	0.050	0.049
GDB 9	0.095	0.095	0.155	0.174	**0.034**	0.095	0.036	0.095	0.049	0.047
GDB 10	0.099	0.099	0.165	0.185	**0.038**	0.099	0.040	0.099	0.055	0.053
GDB 11	0.116	0.116	0.169	0.192	**0.049**	0.116	0.051	0.116	0.065	0.063
GDB 12	0.117	0.117	0.172	0.195	**0.048**	0.117	0.050	0.117	0.065	0.062
GDB 13	0.122	0.122	0.182	0.207	**0.051**	0.122	0.053	0.122	0.068	0.065
S66x8	0.062	0.062	0.129	0.146	**0.031**	0.062	0.033	0.062	0.043	0.040
Tripeptide	0.128	0.128	0.311	0.371	0.073	0.128	**0.070**	0.128	0.079	0.074

Table S36: MAE charge in e for DFTBML and various models on COMP6 benchmark suite

Test set	Auorg	MIO	GFN1-xTB	GFN2-xTB	DFTBML CC	Repulsive CC	DFTBML DFT	Repulsive DFT	Transfer CC	Transfer DFT
Ani MD	0.071	0.071	0.090	0.076	**0.053**	0.071	0.057	0.071	0.058	0.057
Drugbank	0.065	0.065	0.091	0.074	**0.049**	0.065	0.053	0.065	0.052	0.051
GDB 7	0.075	0.075	0.101	0.089	**0.046**	0.075	0.050	0.075	0.054	0.052
GDB 8	0.074	0.074	0.100	0.088	**0.047**	0.074	0.052	0.074	0.055	0.054
GDB 9	0.073	0.074	0.097	0.087	**0.046**	0.073	0.050	0.073	0.053	0.052
GDB 10	0.073	0.074	0.098	0.085	**0.048**	0.073	0.052	0.073	0.055	0.054
GDB 11	0.071	0.071	0.095	0.083	**0.050**	0.071	0.055	0.071	0.056	0.055
GDB 12	0.068	0.068	0.093	0.080	**0.047**	0.068	0.051	0.068	0.052	0.051
GDB 13	0.068	0.068	0.093	0.081	**0.047**	0.068	0.051	0.068	0.053	0.051
S66x8	0.065	0.065	0.094	0.084	**0.045**	0.065	0.048	0.065	0.049	0.047
Tripeptide	0.085	0.085	0.100	0.087	**0.056**	0.085	0.060	0.085	0.064	0.062

Table S37: MAE total energy per heavy atom in kcal/mol for DFTBML and various models on the COMP6 benchmark suite

Test set	Auorg	MIO	GFN1-xTB	GFN2-xTB	DFTBML CC	Repulsive CC	DFTBML DFT	Repulsive DFT	Transfer CC	Transfer DFT
Ani MD	0.27	0.24	0.24	0.43	0.24	0.31	0.20	0.26	0.21	**0.16**
Drugbank	0.68	0.64	0.59	0.57	0.39	0.54	**0.31**	0.48	0.41	0.32
GDB 7	1.33	1.29	1.20	1.09	0.55	0.89	**0.45**	0.81	0.62	0.49
GDB 8	1.28	1.24	1.12	1.01	0.51	0.80	**0.41**	0.74	0.57	0.44
GDB 9	1.22	1.18	1.00	0.96	0.49	0.82	**0.38**	0.73	0.55	0.41
GDB 10	1.07	1.04	0.98	0.92	0.46	0.72	**0.37**	0.65	0.53	0.40
GDB 11	1.15	1.14	0.94	0.97	0.53	0.80	**0.37**	0.72	0.61	0.41
GDB 12	1.14	1.13	0.88	0.92	0.51	0.80	**0.35**	0.70	0.56	0.37
GDB 13	1.08	1.07	0.87	0.88	0.50	0.78	**0.34**	0.68	0.56	0.36
S66x8	0.70	0.58	0.64	0.65	0.41	0.74	**0.41**	0.71	0.58	0.44
Tripeptide	0.35	0.35	0.24	0.28	0.19	0.30	**0.15**	0.24	0.24	0.18

Table S38: MAE total energy per atom in kcal/mol for DFTBML and various models on the COMP6 benchmark suite

Test set	Auorg	MIO	GFN1-xTB	GFN2-xTB	DFTBML CC	Repulsive CC	DFTBML DFT	Repulsive DFT	Transfer CC	Transfer DFT
Ani MD	0.13	0.12	0.12	0.21	0.11	0.15	0.09	0.12	0.10	**0.07**
Drugbank	0.35	0.33	0.30	0.29	0.20	0.27	**0.16**	0.24	0.21	0.16
GDB 7	0.66	0.64	0.59	0.56	0.27	0.43	**0.22**	0.39	0.30	0.25
GDB 8	0.64	0.62	0.56	0.52	0.26	0.39	**0.21**	0.36	0.29	0.22
GDB 9	0.60	0.59	0.49	0.49	0.24	0.40	**0.19**	0.36	0.28	0.21
GDB 10	0.54	0.52	0.49	0.47	0.23	0.36	**0.19**	0.33	0.27	0.20
GDB 11	0.56	0.55	0.46	0.48	0.26	0.39	**0.18**	0.35	0.30	0.20
GDB 12	0.56	0.55	0.43	0.45	0.25	0.39	**0.17**	0.34	0.27	0.18
GDB 13	0.52	0.52	0.42	0.42	0.24	0.37	**0.16**	0.33	0.27	0.18
S66x8	0.28	0.23	0.27	0.27	**0.16**	0.30	0.17	0.29	0.23	0.18
Tripeptide	0.18	0.18	0.12	0.14	0.10	0.15	**0.08**	0.12	0.12	0.09

S14 Analysis of forces

Even though DFTBML is not trained explicitly to forces, the model improves upon forces compared to existing parameterizations of DFTB as shown in Figure S17. Note that for the ANI-1CCX dataset, only forces computed at a double-zeta (DZ) or triple-zeta (TZ) DFT level are available. Based on the computed MAE values, we see that for predicting DFT DZ forces, DFTBML DFT improves over Auorg by 40% while DFTBML CC improves over Auorg by 38%. The percent improvement is greater when comparing predictions for DFT TZ forces, where DFTBML DFT improves over Auorg by 48% and DFTBML CC improves over Auorg by 39%. This likely results from the DFTBML DFT model being trained to energies from DFT TZ. Similar percentage improvements are seen when comparing MAE values on force magnitudes (Figure S18). For both components and magnitudes, training of the parameters both narrows the distribution, towards a better correlation of predicted and target forces, and reduces systematic errors seen in the distributions of the Auorg forces. Although training to energies does improve force predictions, as discussed in the main manuscript, the percent improvement in forces is smaller than the percent improvement seen for energy. This suggests that training directly on forces may lead to improved models.

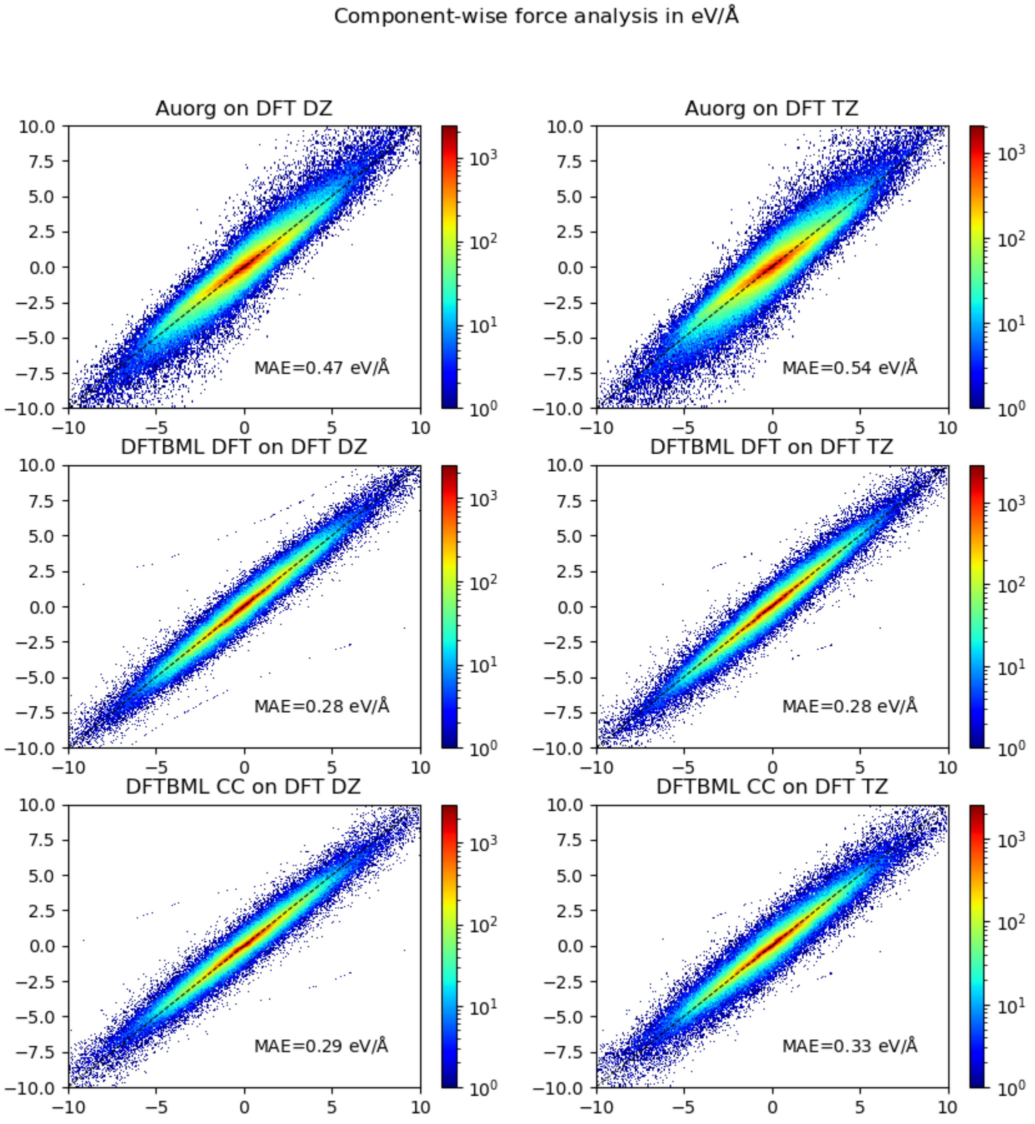

Figure S17: Two-dimensional histogram of component-wise force errors on the standardized ANI-1CCX test set on both double-zeta (DZ) and triple-zeta (TZ) DFT forces.

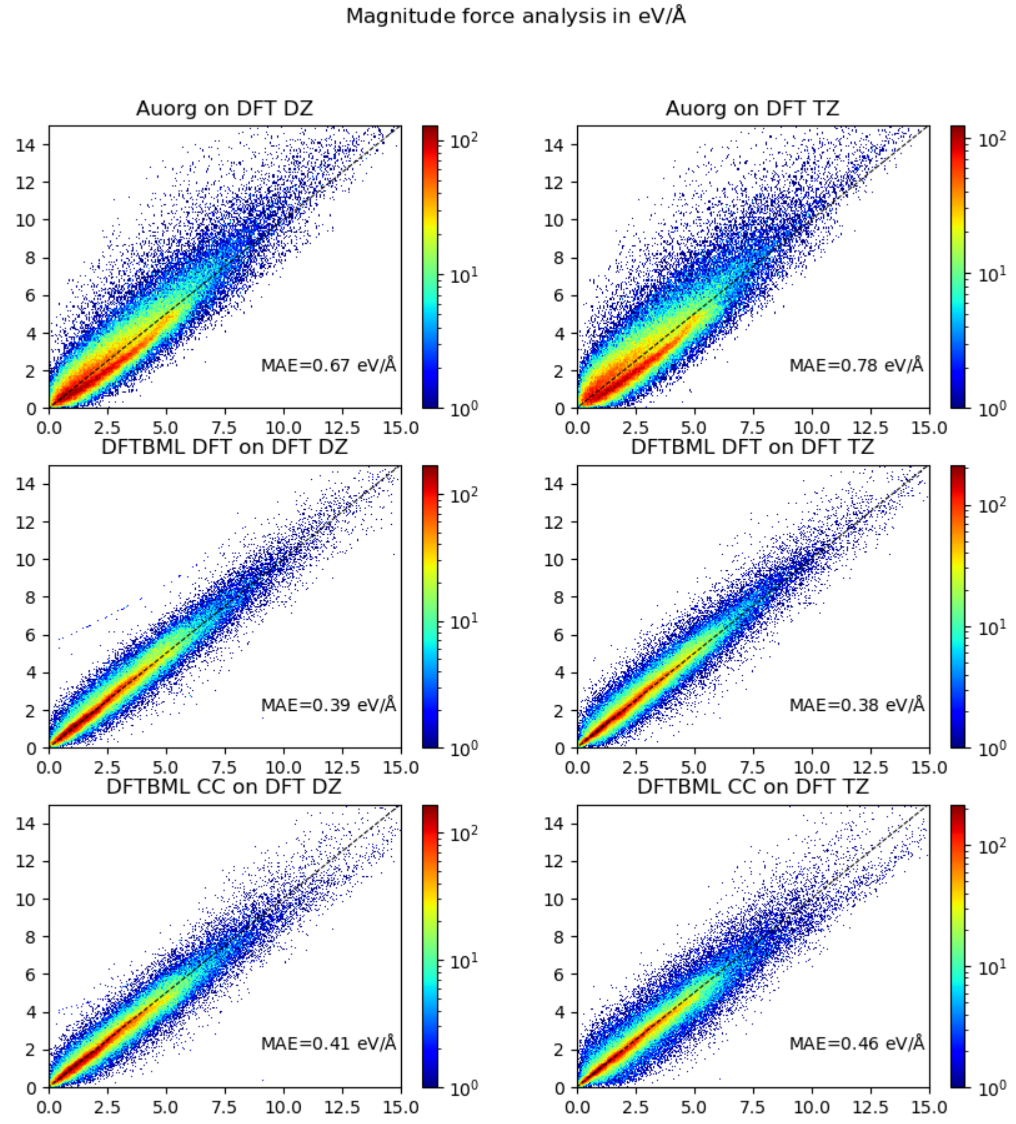

Figure S18: Two-dimensional histogram of force magnitude errors on the standardized ANI-1CCX test set on both double-zeta (DZ) and triple-zeta (TZ) DFT forces.

References

(S1) Elstner, M.; Porezag, D.; Jungnickel, G.; Elsner, J.; Haugk, M.; Frauenheim, T.; Suhai, S.; Seifert, G. Self-consistent-charge density-functional tight-binding method for simulations of complex materials properties. *Phys. Rev. B* **1998**, *58*, 7260–7268.

(S2) Elstner, M.; Seifert, G. Density functional tight binding. *Philosophical Transactions of the Royal Society A: Mathematical, Physical and Engineering Sciences* **2014**, *372*, 20120483.

(S3) Li, H.; Collins, C.; Tanha, M.; Gordon, G. J.; Yaron, D. J. A Density Functional Tight Binding Layer for Deep Learning of Chemical Hamiltonians. *J. Chem. Theory Comput.* **2018**, *14*, 5764–5776.

(S4) Vandenberghe, L. The CVXOPT linear and quadratic cone program solvers. *https://cvxopt.org/index.html* **2010**,

(S5) Seeger, M.; Hetzel, A.; Dai, Z.; Meissner, E.; Lawrence, N. D. Auto-Differentiating Linear Algebra. *NIPS 2017 Workshop Autodiff* **2017**,
